# The Impact of Maltodextrin and Inulin on the Protection of Natural Antioxidants in Powders Made of Saskatoon Berry Fruit, Juice, and Pomace as Functional Food Ingredients

**DOI:** 10.3390/molecules25081805

**Published:** 2020-04-15

**Authors:** Sabina Lachowicz, Anna Michalska-Ciechanowska, Jan Oszmiański

**Affiliations:** 1 Department of Fermentation and Cereals Technology, Faculty of Biotechnology and Food Science, Wrocław University of Environmental and Life Sciences, 51–630 Wrocław, Poland; 2 Department of Fruit, Vegetable and Plant Nutraceutical Technology, Faculty of Biotechnology and Food Science, Wrocław University of Environmental and Life Sciences, 51–630 Wrocław, Poland; anna.michalska@upwr.edu.pl (A.M.-C.); jan.oszmianski@upwr.edu.pl (J.O.)

**Keywords:** *Amelanchier alnifolia* Nutt., carriers, powders, bioactive compounds, functional food

## Abstract

The objective of this study was to examine the effect of inulin and maltodextrin applied during vacuum drying of Saskatoon berry fruit, juice, and pomace on the retention of bioactive compounds and antioxidant capacity (radical scavenging capacity (ABTS), ferric reducing antioxidant potential (FRAP)) of powders obtained. Ultra-high performance liquid chromatography (UPLC-PDA-ESI-MS/MS) was used to identify major groups of polyphenolic compounds, such as: flavan-3-ols (35% of all polyphenols for fruit powder, 33% for juice powder, and 39% for pomace powders of all polyphenols), anthocyanins (26% for fruit powder, 5% for juice powder, and 34% for pomace), phenolic acids (33% for fruit powder, 55% for juice powder, and 20% for pomace powder), and flavanols (6% for fruit powder, 6% for juice powder, and 7% for pomace powder). In general, the content of polyphenols was more dependent on the content than on the type of carrier used for drying, regardless of the matrix tested. The average sum of polyphenols and the antioxidant activity (for ABTS and FRAP assay) of the powders with 30% of carrier addition were 5054.2 mg/100 g dry matter (d.m.) as well as 5.3 and 3.6 mmol Trolox/100 g d.m. in the ABTS and FRAP tests, respectively. The increase in carrier concentration by 20% caused a decrease of 1.5-fold in the content of polyphenols and a 1.6-fold and 1.5-fold in the antioxidant potential, regardless of the matrix tested. The principal component analysis (PCA) analysis indicated that the freeze-drying process led to the lowest degradation of the identified compounds, regardless of the matrix tested, with the exception of juice and pomace powders dried by vacuum drying at 60 °C. In this case, the release of (−)-epicatechin was observed, causing an increase in the flavanol contents. Thus, this work demonstrated the effect of processing and matrix composition on the preservation of antioxidant bioactives in Saskatoon berry powders. Properly designed high-quality Saskatoon berry powders with the mentioned carriers may be used as nutraceutical additives to fortify food products and to improve their functional properties.

## 1. Introduction

Saskatoon berry (*Amelanchier alnifolia* Nutt.) belongs to the *Rosaceae* family and is commonly found in the North America [1,2], and also in Poland. In folk medicine, it was used to combat many ailments [3]. Its fruits contain polyphenolic compounds, like e.g., anthocyanins, phenolic acids (mainly hydroxycinnamic acids), polymers of procyanidins, (+)-catechins, (−)-epicatechins, and quercetin derivatives [3,4]. These compounds have a wide spectrum of biological functions and biochemical activities, e.g., anti-diabetic, anti-inflammatory, antioxidant, and anti-cancer properties [1,3,5,6].

In order to preserve of bioactive constituents from fruits during processing, the powdering process has recently been proposed as one of the possible ways to obtain products with natural health-promoting constituents. The processing of fruits, including thermal treatment, causes significant changes in the composition of processed products compared to the raw materials [7]. The transformation of fruit material into powdered is made easier when the addition of a carrier agent is applied. Such an approach may lead to stability improvement of natural bioactive compounds during further processing. This may allow entrapping polyphenolic compounds in the carrier shell or matrix so that the thermolabile active substance gains protection against degradation and their stability [8,9,10]. The most commonly used carriers include maltodextrin, gums, proteins, and starches [11,12]. Recently, carriers with functional properties have been used during food the powdering process. In this study, we used two carriers: maltodextrin—because it is the most commonly used carrier, and inulin—due to its proven prebiotic effects and pro-health potential. What is more, the addition of maltodextrin and inulin improve the drying yield [13,14].

The selection of carriers, their concentration, and drying technique are all important factors that affect the profile and concentration of antioxidant compounds. Although the impact of carriers and their concentration on the profile of bioactive compounds in fruit powders has been evaluated in some previous studies [15,16], there is lack of information on the impact of these factors on the phytochemicals of *Amelanchier alnifolia* Nutt. Thus, the objective of the present study was to investigate the influence of maltodextrin and inulin used as a carrier and of their concentration on the quantitative and qualitative composition of polyphenolic profile of *Amelanchier alnifolia* Nutt. fruit, juice, and pomace powders obtained by freeze-drying and vacuum-drying at 50 and 60 °C. Polyphenol profile was determined by ultra-high performance liquid chromatography (UPLC-PDA-ESI-MS/MS) and their antioxidant properties determined by radical scavenging capacity (ABTS), and ferric reducing antioxidant potential (FRAP).

## 2. Results and Discussion

### 2.1. Polyphenolic Content and Chemical Profile

In the present study, polyphenolic compounds were determined using UPLC-PDA-ESI-MS/MS in Saskatoon berry powders with carriers considering the type of the material subjected to drying (whole fruit, juice, pomace), the drying method, and the type of carrier and its concentration (Table 1 and Tables S1–S4). Detailed analyses allowed identifying 42 compounds classified to four main groups as anthocyanins, phenolic acids, flavonols, and flavan-3-ols. Their presence in powders obtained from Saskatoon berry fruits was also confirmed in earlier works [1,2,4,6,17,18].

The most abundant group belonging to polyphenolic compounds was represented in the analyzed powders by flavan-3-ols (monomers, oligomers, and polymeric procyanidins). These compounds were previously identified in fruits by Lachowicz et al. [6,17]. This group accounted for 35% of total polyphenolic compounds in fruit powder, for 33% in juice powder, and for 39% in pomace powder. Among the monomers and oligomers, the presence of 14 compounds was identified; they accounted for 36% of total flavan-3-ols in fruit powder, for 67% in juice powder, and for 20% in pomace powder. In addition, the presence of polymeric procyanidins was determined by phloroglucinolysis (they accounted for 64% of total flavan-3-ols in fruit powder, for 33% in juice powder, and for 80% in pomace powder) (Table S1). In this research two monomers (main ion at *m/z* 289), two dimers (main ion at *m/z* 577), five trimers (main ion at *m/z* 863), and five tetramers (main ion at *m*/*z* 1153) were identified based on MS data and available literature [1,17].

Anthocyanins were the second abundant group determined in the powders [1] and constituted 26% of total polyphenolic compounds in fruit powder, 5% in juice powder, and 34% in pomace powder. Furthermore, six cyanidins were identified including four glycosides of cyanidins (cyanidin-3-*O*-galactoside: *m/z* 449, -3-*O*-glucoside: *m/z* 499, -3-*O*-xyloside: *m/z* 419, -3-*O*-arabinoside: *m/z* 419) and two cyanidin aglycons (*m/z* 287). Among the 6 identified compounds, the major compound belonging to anthocyanins was cyanidin-3-*O*-galactoside (it accounted for 69.9% of all anthocyanins). This was confirmed in earlier reports [2].

Phenolic acids were the next group of polyphenols that accounted for 33% of total polyphenolic compounds in fruit powder, for 55% in juice powder, and for 20% in pomace powder. According to the MS spectra, standards, and literature, eight hydroxycinnamic acids were determined, including: three isomers of caffeoylquinic acid (*m/z* 353), two coumaroylquinic acids (*m/z* 353), and three isomers of caffeic acids (*m/z* 341), as well as one hydroxybenzoic acid identified as protocatechuic acid (*m/z* 153). The major phenolic acids were 5-*O*-caffeoylquinic acid (56% of all phenolic acids), and 4-*O*-caffeoylquinic acid (19%). The mass noted on the negative mode was *m/z* 353 with similar MS spectra to those reported in literature [1,2].

The last and the smallest group of phenolic compounds was constituted by flavonols [17]. This group accounted for 6% of total flavonols in fruit powder, for 6% in juice powder, and for 7% in pomace powder; and the major compound was quercetin-3-*O*-galactoside [1,2] which represented 46% of all flavonols. In this work, thirteen flavonols were identified including ten glycosides of kaempferol and quercetin (based on ion at *m/z* 285 and 301, respectively), two acetylated compounds (*m/z* 507), and one quercetin aglycon (*m/z* 301) [1,2].

The average total content of identified polyphenols was 6535 mg/100 g d.m. in pomace powders. Their content was lower by 38% and 70% in fruit and juice powders, respectively (Table 1 and Tables S1–S4). This is due to the accumulation location of these constituents in the outer layer of the fruit that remains in the pomace during pressing [19,20]. In addition, the highest antioxidant capacity was found in pomace powders and was 3.4-fold (ABTS assay) and 3-fold (FRAP assay) higher when compared to the fruit powders as well as 7-fold (ABTS assay) and 6-fold (FRAP assay) compared to juice powders (Table 2). These relationships were also confirmed during the drying of chokeberry [21] and cranberry fruits [22] and can be explained by distribution of phenols, because they are not equally distributed in the plant material [22]. Anthocyanins and flavonols are mostly presented in the peel of berry, also polymeric procyanidins, monomers, and oligomers of flavan-3-ols are essentially located in the peel, but some content of these compounds occur in the pulp thus in the juice [21,22]. In addition, conditions of juice processing, mash maceration, and the type of enzymatic preparation may significantly impact the content of polyphenols in the juice and pomace of product [21,22]. This fact is widely known in the literature, but there is little information on the impact of carriers and their quantity and drying methods on the individual matrix, especially of Saskatoon berry. What is more, according to literature [1,2,3,17,23,24], the Saskatoon berry fruits are an excellent source of polyphenolic compounds and antioxidant properties responsible for their proved anti-inflammatory, antidiabetic, and chemo-protective effects. They have more bioactive compounds than e.g., blueberry, blackberry, bilberry, raspberry, and strawberry [25,26]. However, little is known about the use of this raw material as a functional additive. Available data relate only to Saskatoon berry supplements and used the accumulation of plasma levels of anthocyanins [27]. *Amelanchier alnifolia* Nutt. (90 g) were administered immediately after every low antioxidant meal. The authors noted plasma concentrations of cyanidin-3-*O*-xyloside and cyanidin-3-*O*-galactoside were significantly increased following 3 consecutive supplements 4 h apart. Therefore, they recommend that anthocyanin supplements with every meal would higher their plasma concentrations [27]. Another work concerned the prevention of diabetes in obese mice by consuming Saskatoon berry [28]. Male mice were served a control diet, high fat and high sucrose diet, and high fat and high sucrose with 5% of Saskatoon berry powder (weight/weight) diet for 15 weeks. The results showed that Saskatoon berry powder suppressed high fat, high sucrose diet-induced hyperglycemia, hyperlipidemia, insulin resistance, and vascular inflammation [28]. Therefore, research was undertaken to obtain new functional additives (powders) from various Saskatoon berry matrices with the highest possible content of antioxidant compounds.

### 2.2. Influence Type of Carriers on Polyphenolic Compounds and Antioxidant Capacity in Dependence on the Matrix

**Figure 1:**
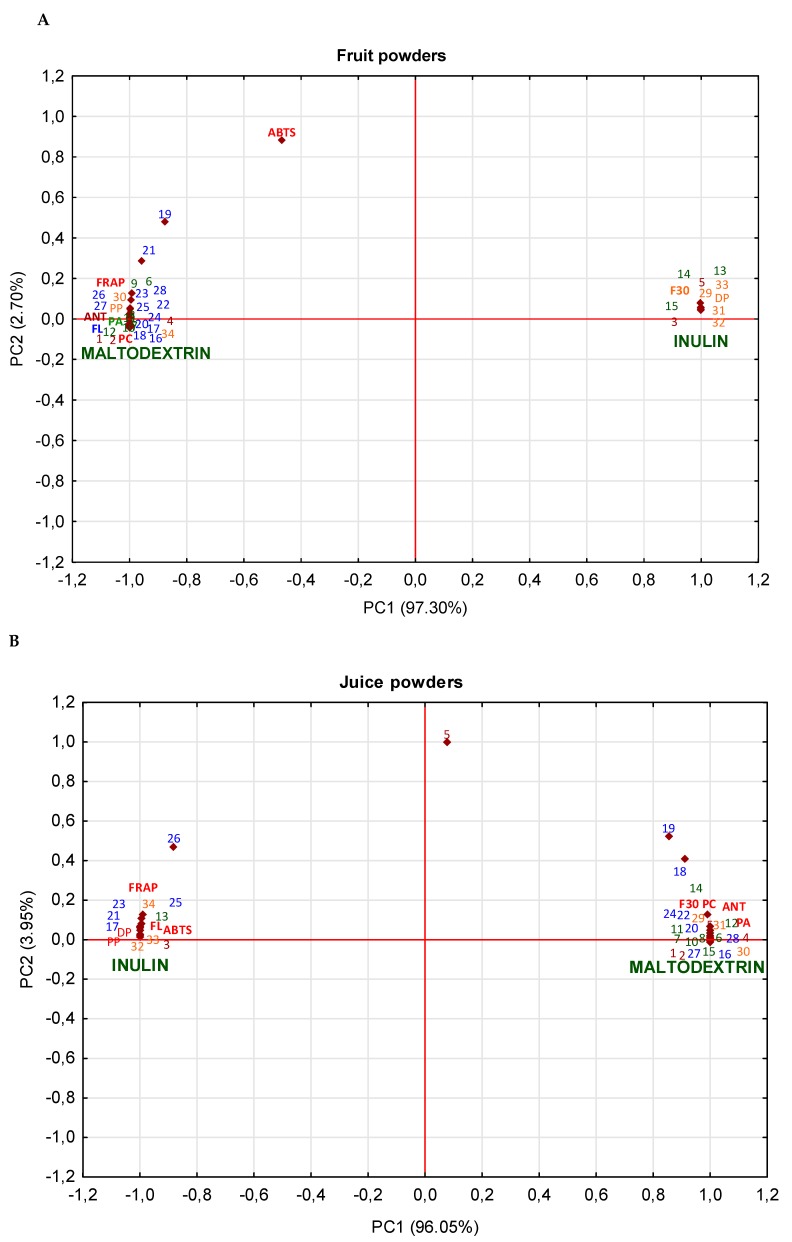

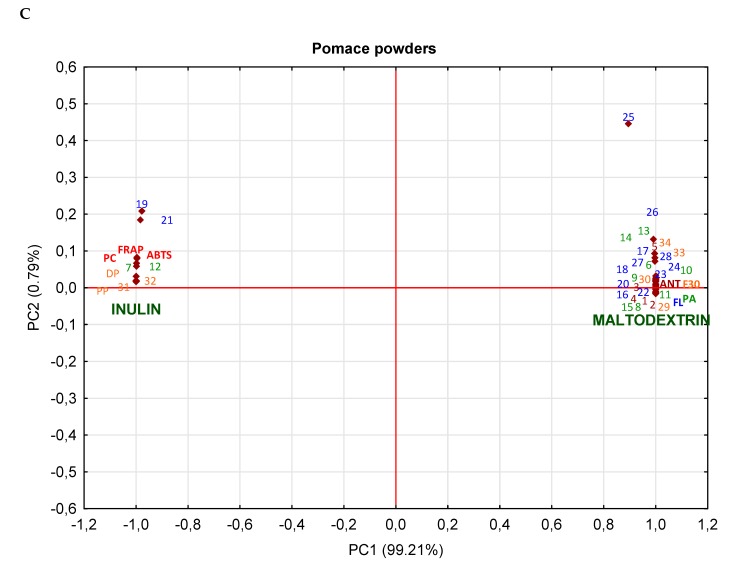
Principal component analysis (PCA) of the impact of type of carrier to phytochemical parameter in fruit (**A**), juice (**B**), pomace (**C**) powders. *Abbreviations:* ANT—sum of anthocyanins; FL—sum of flavonols; PA—sum of phenolic acid; PP—polymeric procyanidins; F3O—sum of flavan-3-ols (monomers and oligomers); DP—degree of polymerization; PC—sum of polyphenolic compounds; **1**—cyanidin-3-*O*-galactoside; **2**—cyanidin-3-*O*-glucoside; **3**—cyanidin-3-*O*-arabinoside; **4**—cyanidin-3-*O*-xyloside; **5**—cyanidin; **6**—protocatechuic acid; **7**—caffeic acid glucoside; **8**—caffeoylhexose; **9**—acid isomers; **10**—3-*O*-caffeoylquinic acid; **11**—5-*O*-caffeoylquinic acid; **12**—4-*O*-caffeoylquinic acid; **13**—3-*O*-*p*-coumaroylquinic acid; **14**—di-caffeoylquinic acid; **15**—di-caffeoylquinic acid; **16**—kaempferol-3-*O*-galactoside; **17**—quercetin-3-*O*-arabinobioside; **18**—kampferol-3-*O*-glucoside; **19**—quercetin; **20**—quercetin-3-*O*-rutinoside; **21**—quercetin-3-*O*-robinobioside; **22**—quercetin-3-*O*-galactoside; **23**—quercetin-3-*O*-glucoside; **24**—quercetin-3-*O*-arabinoside; **25**—quercetin-3-*O*-xyloside; **26**—quercetin-3-*O*-(6’’-acetyl)glucoside; **27**—quercetin-3-*O*-(6’’-acetyl)galactoside; **28**—quercetin-deoxyhexo-hexoside; **29**—sum of B-type procyanidin tetramer; **30**—sum of B-type procyanidin trimer; **31**—sum of B-type procyanidin dimer; **32**—A-type procyanidin dimer; **33**—(-)-epicatechin; **34**—(+)-catechin.

The principal component analysis (PCA) was used to study the impact of the type of carriers on the protection of polyphenolic compounds and their antioxidant activity depending on the matrix tested (Figure 1). It showed that in the case of fruit powders, drying in the presence of inulin was associated with a higher concentration and protection of flavan-3-ols group and their individual compounds ((+)-catechin, B-type procyanidin: dimer, and tetramer, and A-type procyanidin dimer), 3 and 4-*O*-caffeoylquinic acids, di-caffeoylquinic acid, and cyanidin-3-*O*-arabinoside. While maltodextrin caused the higher retention and ensured protection of polymeric procyanidins, total phenolic acids and their derivatives, total flavonols and their derivatives, total anthocyanins and their derivatives, and total phenolic acids and their antioxidant activity (strong correlation with the ferric reducing antioxidant potential (FRAP)) and positive correlation with the radical scavenging capacity (ABTS)). Bakowska-Barczak and Kołodziejczyk [29] also reported that the use of maltodextrin resulted in the highest contents of anthocyanins and polyphenols in black currant powders. The higher retention of polyphenolic compounds was also noted in cactus pear with maltodextrin when compared to inulin [30]. In turn, PCA analysis showed that in the case of juice powders, inulin had a stronger impact on total flavonols and their main derivatives, (+)-catechin, (−)-epicatechin, B-type procyanidin dimer, polymeric procyanidins, cyanidin-3-*O*-arabinoside, and 3-*O*-caffeoylquinic acid; while, maltodextrin led to the higher retention of total phenolic acid, total anthocyanins, total flavan-3-ols. What is more, the ability to scavenge the ABTS radicals was higher in the case of the juice powders with maltodextrin addition, whereas higher FRAP values were noted for the powders prepared with inulin. The addition of maltodextrin into juice powders affected the release of free cyanidin and quercetin aglycons. Whereas, in black currant juice powder, the use of inulin resulted in a 9% higher anthocyanin retention compared to the use of maltodextrin [16]. Furthermore, the PCA analysis of pomace powders showed that the product with inulin had higher contents of total of polyphenolic compounds, A and B-type procyanidin dimer, polymeric procyanidin, and quercetin-3-*O*-robinobioside, and also a higher degree of polymerization. This product had higher antioxidant potency, while maltodextrin was responsible for the protection of total anthocyanins and their derivatives, total flavan-3-ols and their derivatives, total phenolic acids and their derivatives, and total flavonols and their derivatives. Thus, the type of carrier and its impact on antioxidant compounds depended mainly on the analyzed matrix. The high antioxidant power of the juice and pomace powders with inulin and also of the fruit powders with maltodextrin can be affected by polymerized compounds. This can also be confirmed by a strong correlation between the polymerized compounds and antioxidant activity i.e., *r* = 0.997 for FRAP and 0.430 for ABTS (for fruit powders), *r* = 0.995 and 0.998 for FRAP and ABTS assay (juice powders), and *r =* 0.997 and 0.998 for FRAP and ABTS assay (pomace powders). Therefore, the choice of a carrier will largely depend on the desired composition of bioactive compounds with antioxidant activity for the functional additive regardless of the Saskatoon berry matrix tested.

### 2.3. Impact of the Amount of Added Carriers’ on Polyphenols Content and Antioxidant Capacity in Dependence on the Matrix

**Figure 2:**
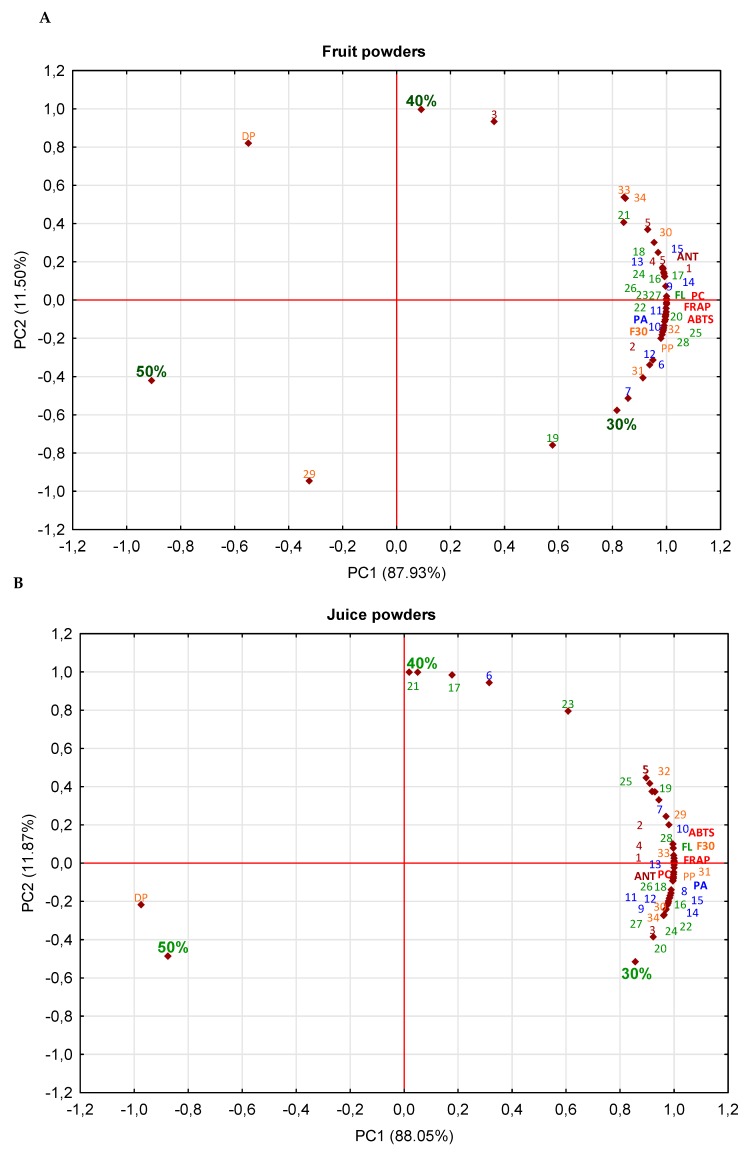

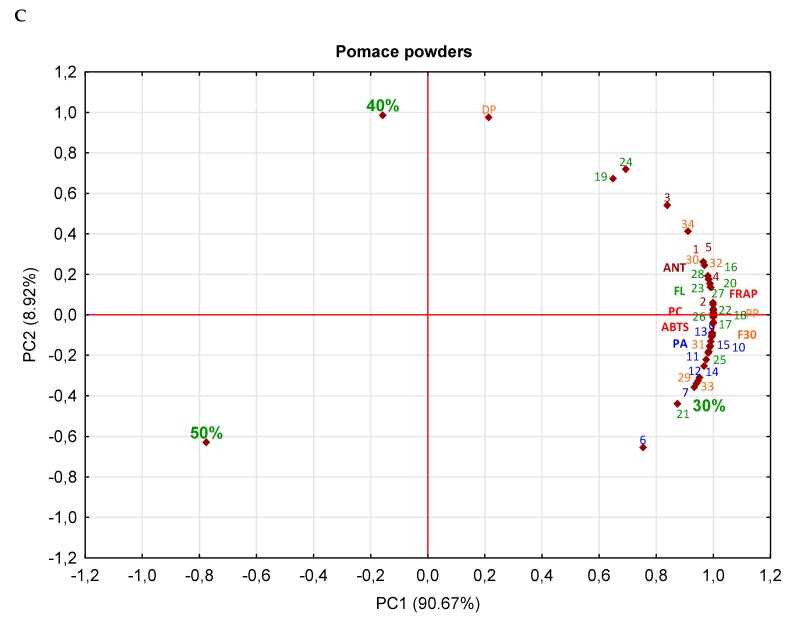
Principal component analysis (PCA) of the impact of carriers’ concentration on phytochemicals in fruit (**A**), juice (**B**), pomace (**C**) powders. *Abbreviations:* 30%, 40%, 50%, concentration; ANT—sum of anthocyanins; FL—sum of flavonols; PA—sum of phenolic acid; PP—polymeric procyanidins; F3O—sum of flavan-3-ols (monomers and oligomers); DP—degree of polymerization; PC—sum of polyphenolic compounds; **1**—cyanidin-3-*O*-galactoside; **2**—cyanidin-3-*O*-glucoside; **3**—cyanidin-3-*O*-arabinoside; **4**—cyanidin-3-*O*-xyloside; **5**—cyanidin; **6**—protocatechuic acid; **7**—caffeic acid glucoside; **8**—caffeoylhexose; **9**—trihydroxycinnamoylquinic acid isomers; **10**—3-*O*-caffeoylquinic acid; **11**—5-*O*-caffeoylquinic acid; **12**—4-*O*-caffeoylquinic acid; **13**—3-*O*-*p*-coumaroylquinic acid; **14**—di-caffeoylquinic acid; **15**—di-caffeoylquinic acid; **16**—kaempferol-3-*O*-galactoside; **17**—quercetin-3-*O*-arabinobioside; **18**—kaempferol-3-*O*-glucoside; **19**—quercetin; **20**—quercetin-3-*O*-rutinoside; **21**—quercetin-3-*O*-robinobioside; **22**—quercetin-3-*O*-galactoside; **23**—quercetin-3-*O*-glucoside; **24**—quercetin-3-*O*-arabinoside; **25**—quercetin-3-*O*-xyloside; **26**—quercetin-3-*O*-(6’’-acetyl)glucoside; **27**—quercetin-3-*O*-(6’’-acetyl)galactoside; **28**—quercetin-deoxyhexo-hexoside; **29**—sum of B-type procyanidin tetramer; **30**—sum of B-type procyanidin trimer; **31**—sum of B-type procyanidin dimer; **32**—A-type procyanidin dimer; **33**—(-)-epicatechin; **34**—(+)-catechin.

The concentration of carriers had a stronger impact on the retention of polyphenolic compounds than the type of carrier or drying method. A similar observation was made for black currant powders with the use of the mentioned-above carriers for drying [16]. In turn, addition of maltodextrin at the level of 15% was found to protect the bioactive compounds from thermal degradation [31]. The principal component analysis (PCA) was used to evaluate the influence of the concentration of carriers on the protection of antioxidant compounds depending on the matrix tested (Figure 2). PCA graph for powders with 30% of carriers showed by 20% and 37% higher content of total polyphenols, and by 29% and 47% higher antioxidant potential determined with ABTS and FRAP assays compared to the powders obtained with 40% and 50% of carriers, respectively, regardless of the matrix tested. Probably, the content of phenolics was affected by the dilution effect with the carrier; the higher the carrier concentration, the lower the content of bioactive compounds. A similar observation was made in the case of *Eugenia dysenterica* DC. powders pointing to a concentration of a carrier on the content of polyphenols. Samples obtained using 10% of carriers showed the highest content of polyphenols, while the powders with 30% of carrier presenting the lowest amount of phenols [32]. This can also be confirmed by a strong positive correlation between individual polyphenolic compounds (anthocyanins, phenolic acids, flavonols, flavan-3-ols) and antioxidant activity (ABTS assay) of *r* = 0.979, 0.999, 0.998, 0.992 (for fruit powders), *r* = 0.993, 0.974, 0.998, 0.999 (juice powders), and *r* = 0.958, 0.993, 0.994, 0.998 (pomace powders). Depending on the matrix, the lowest loss of polyphenolic compounds was noted in juice powders and it was 17% and 21% with 40% and 50% of carriers compared to the powders with 30% of carriers. While in fruit and pomace powders losses were 11% and 18% higher. Greater degradation of flavan-3-ols (monomers and oligomer) and polymeric procyanidin was found in the fruit and pomace powders than in the juice powders; it reached ca. 24% and 30%. As for the anthocyanins, the greatest degradation was found in fruit powder (ca. 43% in product with 50% of carrier) > pomace powder (around 40%) > juice powders (38%). While, the smallest degradation of phenolic acids and flavonols was found in the pomace powders—around 15% and 26% and 12% and 28% in products with 40% and 50% of carriers, respectively, compared to the final product with 30% of carrier. Probably high stability can be due to presence of cell wall polysaccharide compounds e.g., pectins, whether lower water activity [21,22]. Additionally, it might be due to the better encapsulation process of these samples, which is confirmed by Daza et al. [32] who followed the highest retentions of bioactive compounds in cagaita powders with 20%–30% of carriers. However, the higher carrier concentration affected the protection of individual compounds, e.g., in the case of fruit powders—40% of carrier influenced higher protection of cyanidin-3-O-arabinose and a higher degree of polymerization, but 50% of carrier affected the B-type procyanidin tetramer. Whereas in the case of juice powders—40% of carrier caused high retention of quercetin-3-O-robinobioside, -3-O-arabinoside, -3-O-glucoside, and protocatechuic acid, but 50% of carrier caused a high degree of polymerization. Finally, in the case of pomace powders—40% of carrier caused a high degree of polarization just like in the fruit powders. Indicating that might have led to a release of the compounds from more polymerized structures. Similarly as with pulp and juice powders made from cagaita and plum, the concentration of carriers had a strong influence on the decrease of total polyphenolic compounds [32,33]. Thus, the higher carrier concentration led to a lower polyphenol content and lower antioxidant activity, indicating the strong effect of the initial composition of the matrix tested. Moreover, dried products with carriers showed a 1.6 and 1.9, and 1.5 times higher protection of the antioxidant capacity and phenolic compounds, than the products without carriers [17]. Which is also confirmed by other authors who confirmed that the use of carriers affects the protection of bioactive compounds [32,33]. Different interactions between the powders compounds and the carrier might also occur compared to the powders without a carrier [32].

### 2.4. Impact of the Type of Drying Method on Polyphenolic Compounds and Antioxidant Capacity in Dependence on the Matrix

**Figure 3:**
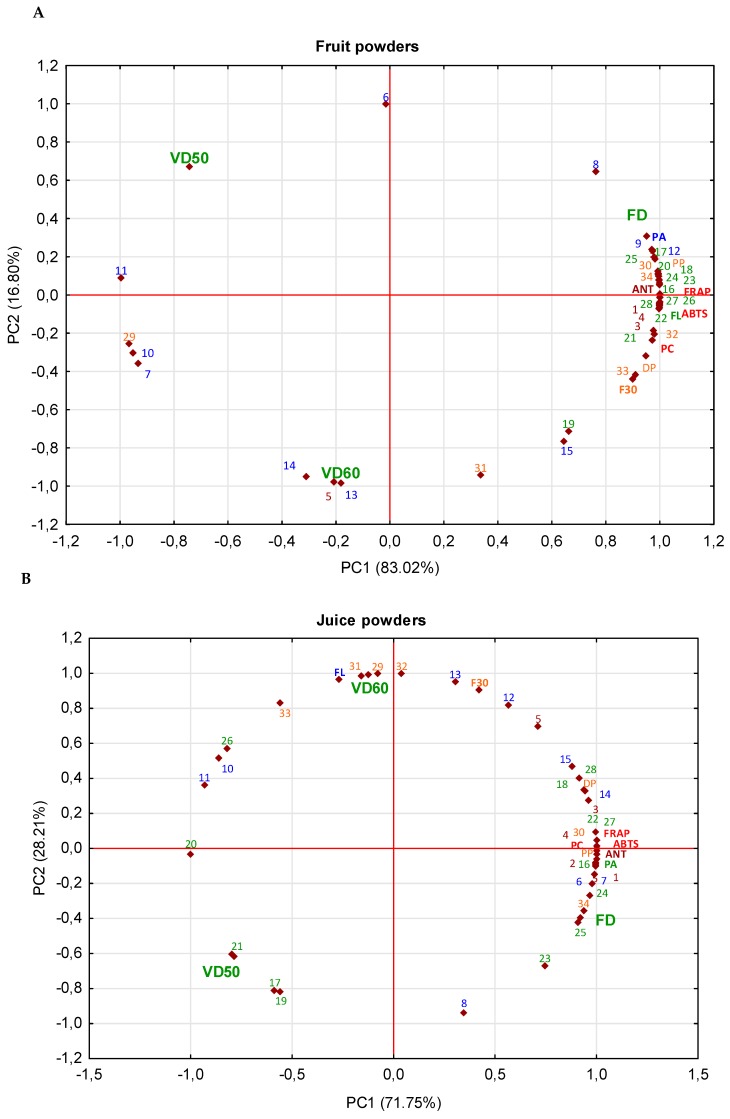

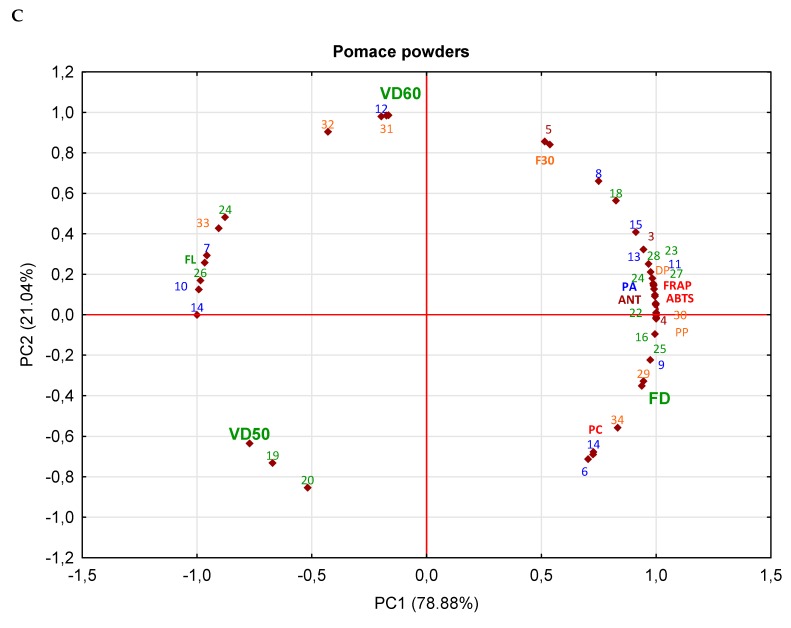
Principal component analysis (PCA) of the impact of drying method on phytochemicals in fruit (**A**), juice (**B**), pomace (**C**) powders. *Abbreviations:* FD, freeze-drying; VD/50, vacuum-drying in 50 °C; VD/60, vacuum-drying in 60 °C; ANT—sum of anthocyanins; FL—sum of flavonols; PA—sum of phenolic acid; PP—polymeric procyanidins; F3O—sum of flavan-3-ols (monomers and oligomers); DP—degree of polymerization; PC—sum of polyphenolic compounds; **1**—cyanidin-3-*O*-galactoside; **2**—cyanidin-3-*O*-glucoside; **3**—cyanidin-3-*O*-arabinoside; **4**—cyanidin-3-*O*-xyloside; **5**—cyanidin; **6**—protocatechuic acid; **7**—caffeic acid glucoside; **8**—caffeoylhexose; **9**—trihydroxycinnamoylquinic acid isomers; **10**—3-*O-*caffeoylquinic acid; **11**—5*-O*-caffeoylquinic acid; **12**—4*-O*-caffeoylquinic acid; **13**—3-*O*-*p*-coumaroylquinic acid; **14**—di-caffeoylquinic acid; **15**—di-caffeoylquinic acid; **16**—kaempferol-3-*O*-galactoside; **17**—quercetin-3-*O*-arabinobioside; **18**—kampferol-3-*O*-glucoside; **19**—quercetin; **20**—quercetin-3-*O*-xrutinoside; **21**—quercetin-3-*O*-robinobioside; **22**—quercetin-3-*O*-galactoside; **23**—quercetin-3-*O*-glucoside; **24**—quercetin-3-*O*-arabinoside; **25**—quercetin-3-*O*-xyloside; **26**—quercetin-3-*O*-(6’’-acetyl)glucoside; **27**—quercetin-3-*O*-(6’’-acetyl)galactoside; **28**—quercetin-deoxyhexo-hexoside; **29**—sum of B-type procyanidin tetramer; **30**—sum of B-type procyanidin trimer; **31**—sum of B-type procyanidin dimer; **32**—A-type procyanidin dimer; **33**—(-)-epicatechin; **34**—(+)-catechin.

The PCA analyses showed that the powders produced after the freeze-drying process had the highest content of almost all polyphenolic compounds and antioxidant activity, regardless of the matrix tested (Figure 3). Similar observations were made during drying Averrhoa carambola pomace. The freeze-drying with carriers was more effective in ensuring the retention of polyphenolic compounds when compared to spray drying [12]. Moreover, after freeze-drying Averrhoa carambola pomace, it was noted that the higher the drying process temperature, the lower the antioxidant activity, while opposite observation was made for the juice powder. The higher the vacuum drying temperature, the stronger the degradation of anthocyanins in juice powders made from black currant. However, vacuum drying at 50 °C affected high retained phenolic acids and flavonols [16]. The best effect of drying cranberry products was obtained after spray-drying and freeze-drying, while an increase in aglycon of quercetin after vacuum-drying at 60 °C was observed [31]. Therefore, it can be concluded that the antioxidant activity of the tested material was dependent on the content of polyphenolic compounds, which did not degrade under mild freeze-drying conditions.

Vacuum drying method affected the retention of individual compounds: for fruit powders, vacuum drying at 50 °C resulted in a higher retention of hydroxybenzoic acid (protocatechuic acid), and hydroxycinnamic acid (caffeic acid) in powders whereas drying at 60 °C had an impact on B-type procyanidin dimer, and tetramer, 4, 3-*O*-caffeoylquinic acids, di-caffeoylquinic acid, free cyanidin, and quercetin aglycon. In the case of juice powders, application of the temperature of 50 °C during vacuum drying resulted in a higher retention of quercetin-3-*O*-robinobioside, -3-*O*-arabinoside, and quercetin aglycon. Drying at 60 °C influenced a higher content of total flavonols and their derivatives, flavan-3-ols and their monomers and oligomers, and free cyanidin aglycon. However, in blackcurrant juice powders, the vacuum drying at 50 °C and 70 °C caused a higher retention of anthocyanins, and according to the authors, this could be due to the inactivation of polyphenol oxidase [16]. In the present study, the higher temperature contributed only to the release of free aglycon of anthocyanins. As far as the pomace powders are concerned, the vacuum drying at 50 °C had a strong impact on the content of free aglycon, and quercetin-3-*O*-rutinoside. The drying at 60 °C influenced the retention of monomers ((−)-epicatechin) and oligomers of flavan-3-ols, and total flavonols and their derivatives. On the other hand, among all compounds identified in flavan-3-ols group, (−)-epicatechin content in juice powders was 2.8 times higher after vacuum drying at 60 °C when compared to the freeze-drying. This suggests the temperature-depended release of this compound from polymerized structures. A similar observation was made for blackcurrant juice the vacuum drying of which at 90 °C resulted in 130 times higher (+)-catechin content compared to vacuum drying at 50 °C. The authors concluded that the controlled release of compounds from polymerized structures during drying may be helpful in obtaining the desired content of polyphenolic compounds in the powders [16]. In turn, a similar increase of flavonols aglycon content under the influence of high temperature as a result of the deglycosylation of flavonol glycosides was observed in cranberry products [31,34]. In addition, similarly to cranberry juice powders [31], the temperature influenced the retention of total flavonols in juice and pomace powders made from Saskatoon berry. This can also be confirmed by negative correlation between flavonols and antioxidant activity of *r =* −0.265 for ABTS assay, and *r* = −0.253 for FRAP assay (for juice powders), and *r* = −0.925 for ABTS assay, and *r =* −0.890 for FRAP assay (pomace powders). Moreover, the increased content of flavonols and their derivatives was also observed upon vacuum drying of juice and pomace powders made of black currant at 50 °C and 70 °C [16,35]; however, the authors observed that an increase of temperature above 80 °C and 90 °C decreased flavonols content. Among all of the compounds tested, anthocyanins were the most unstable constituents when the higher temperature of drying was applied. Their average amount after vacuum drying at 50 °C was 42% lower when compared to the freeze-drying. Moreover, the increase of the temperature during vacuum drying by 10 °C caused their 13% loss. Bakowska-Barczak and Kołodziejczyk [29] reported that the higher the temperature during drying black current powder, the stronger the degradation of anthocyanins. Cai and Corke [36] also noted that a higher drying temperature had a stronger effect on amaranthus betacyanins degradation. On the other hand, depending on the matrix tested, the smallest degradation of anthocyanins was noted in the pomace powder after freeze-drying and it was 1.4 and 1.7 times lower when compared to vacuum drying at 50 and 60 °C, respectively. While, degradation of anthocyanins in the fruit and juice powder after vacuum drying at 50 and 60 °C was 56% and 65%. Probably the highest retention of compounds from pomace can be due to lower water activity, and also with location of compounds because most of them are distributed in berry skin which does not lead to their complete degradation during heat conditions [21,22]. Thus, the research confirmed that mild conditions of freeze-drying ensured the protection of anthocyanins, and this was in line with the previous reports showing that the temperature strongly affected their degradation [37,38,39]. Moreover, the higher the drying temperature is, the greater the degradation of the polyphenolic compounds and their antioxidant activity. The temperature of 50 °C influenced the release of quercetin aglycon. Regardless of the matrix tested, the temperature of 60 °C used for vacuum drying resulted in greater retention of phenolic acid derivatives as well as flavan-3-ols monomers ((−)-epicatechin) and oligomers. It could be due to the partial inactivation of polyphenol oxidase, because Siddiq et al. [40] noted that PPO lost about partial inactivation during thermal treatment of blueberry at 55 and 65 °C [40]. Therefore, the selection of drying technique for the preparation of Saskatoon berry powders is highly important when the retention of bioactive compounds is concerned.

## 3. Materials and Methods

### 3.1. Reagents

Acetonitrile, formic acid, methanol, ABTS (2,2′-azinobis(3-ethylbenzothiazoline-6-sulfonic acid), 6-hydroxy-2,5,7,8-tetramethylchroman-2-carboxylic acid (Trolox, Sigma-Aldrich, Steinheim, Germany), 2,4,6-tri(2-pyridyl)-s-triazine (TPTZ), acetic acid, and phloroglucinol were purchased from Sigma-Aldrich (Steinheim, Germany). (−)-Epicatechin, (+)-catechin, chlorogenic acid, di-caffeic quinic acid, procyanidin B2, procyanidin A2, *p*-coumaric acid, keampferol-3-*O*-galactoside, quercetin-3-*O*-galactoside, caffeic acid, cyanidin-3-*O*-galactoside, and cyanidin-3-*O*-glucoside standards were purchased from Extrasynthese (Lyon, France). Acetonitrile for ultra-phase liquid chromatography (UPLC; gradient grade) and ascorbic acid were from Merck (Darmstadt, Germany).

### 3.2. Material

The raw material used in the study included Saskatoon berries (*A. alnifolia* L.) *cv.* ‘Smoky’ that were purchased from the horticultural farm in Wojciechów near Lublin, Poland (51°14′08″N 22°14′41″E). This cultivar was chosen on the basis of the previous research (Lachowicz et al., 2019). The resulting fruit was ground in Thermomix (Wuppertal, Vorkwek, Germany) at 40 °C for 10 min. Then, the juice was extracted on a hydraulic press and centrifuged. Solutions with carrier concentrations of 30%, 40%, and 50% (*w*/*w*) were prepared. The carriers applied to produce powders were maltodextrin DE (20–40) and inulin (Beneo-Orafti, Belgium). The received fruit-carrier, juice-carrier, and pomace-carrier compositions were dried.

### 3.3. Methods

#### 3.3.1. Drying Methods

Freeze-drying (FD, control) (ca. 100 g) was carried out in a freeze dryer (Christ Alpha 1–4 LSC; Osterode am Harz, Germany) for 24 h. The temperature within the drying chamber was −60 °C, while the heating plates had the temperature of 25 °C.

Vacuum drying (VD) was made in VACUCELL 111 ECO LINE vacuum dryer (MMM Medcenter Einrichtungen GmbH, Planegg, Germany) at 50 and 60 °C under pressure of 0.1 mbar for 24, and 16 h, respectively.

All drying experiment were performed in duplicate. The samples obtained were milled by laboratory mill (IKA A.11, Wilmington, NC, US), and vacuum sealed. The powders were kept in a freezer (−20 °C) until extracts’ preparation.

#### 3.3.2. Identification and Quantification of Polyphenols

Polyphenols were extracted from the fruit, juice, and pomace powders in triplicate (*n* = 3) according to Lachowicz et al. [17]. For polyphenolic compounds: wheat bread (1 g) were extracted with 10 mL of mixture containing UPLC-grade methanol (30 mL/100 mL), and acetic acid (1 mL/100 mL of reagent). The extraction was performed twice by incubation for 20 min under sonication (Sonic 6D, Polsonic, Warsaw, Poland) and with occasional shaking. Next, the slurry was centrifuged at 19,000× *g* for 10 min, and the supernatant was filtered through a hydrophilic PTFE 0.20 μm membrane (Millex Samplicity Filter, Merck, Darmstadt, Germany) and used for analysis.

Qualitative (LC-QTOF-MS) and quantitative (UPLC-PDA-FL) analysis of polyphenols (anthocyanins, flavan-3-ols, flavonols, and phenolic acids) was performed as previously described by Lachowicz et al. (2017). Separations of individual polyphenols were carried out using a UPLC BEH C_18_ column (1.7 μm, 2.1 × 100 mm, Waters Corporation, Milford, MA, USA) at 30 °C. The samples (10 μL) were injected, and the elution was completed in 15 min with a sequence of linear gradients and isocratic flow rates of 0.45 mL/min. The mobile phase consisted of solvent A (2% formic acid; *v*/*v*) and solvent B (100% acetonitrile). The program began with isocratic elution with 99% solvent A (0–1 min), and then a linear gradient was used until 12 min, lowering solvent A to 0%; from 12.5 to 13.5 min, the gradient returned to the initial composition (99% A), and then it was held constant to re-equilibrate the column. Characterization of the single components was carried out via the retention time and the accurate molecular masses. Each compound was optimized to its estimated molecular mass [M − H]−/[M + H]+ in the negative and positive mode before and after fragmentation. The data obtained from UPLC-MS were subsequently entered into the MassLynx 4.0 ChromaLynx Application software (Waters Corporation, Milford, MA, USA). On the basis of these data, the software is able to scan different samples for the characterized substances. The runs were monitored at the wavelength of 360 nm for flavonol glycosides. The PDA spectra were measured over the wavelength range of 200–800 nm in steps of 2 nm. The retention times and spectra were compared to those of the pure standard. The calibration curves were run at 360 nm for the standard keampferol-3-*O*-galactoside, quercetin-3-*O*-galactoside, at 320 nm for the standard of chlorogenic, caffeic, di-caffeic quinic, and *p*-coumaric acids, at 520 nm for the standard cyanidin-3-*O*-galactoside and cyanidin-3-*O*-glucoside and at 280 nm for the standard (−) epicatechin, (+)-catechin, procyanidins B2 and A2, at concentrations ranging from 0.05–5 mg/mL (R^2^ = 0.9999). The measurements were performed in triplicates. The results were expressed as mg per 100 g dry matter (d.m.).

#### 3.3.3. Analysis of Proanthocyanidins by Phloroglucinolysis

Direct phloroglucinolysis of samples was performed as described by Lachowicz et al. [17]. Samples were weighed in an amount of 5 mg, then 0.8 mL of the methanolic solution of phloroglucinol (75 g/L) and ascorbic acid (15 g/L) were added. After addition of 0.4 mL of methanolic HCl (0.3 mol/L), the vials were incubated for 30 min at 50 °C with continuous vortexing in a thermo shaker (TS-100, BioSan, Riga, Latvia). The reaction was stopped by placing the vials in an ice bath with the addition of 0.6 mL of the sodium acetate 0.2 mol/L. Next the vials were centrifuged immediately at 20,000× *g* for 10 min at 4 °C. Samples were stored at 4 °C before reverse phase HPLC (RP-HPLC) analysis. All incubations were done in triplicate. Phloroglucinolysis products were separated on a Cadenza CD C18 (75 mm × 4.6 mm, 3 μm) column (Imtakt, Kyoto, Japan). The liquid chromatograph was a Waters (Milford, MA, USA) system equipped with diode array and scanning fluorescence detectors (Waters 474) and autosampler (Waters 717 plus). Solvent A (25 mL acetic acid and 975 mL water) and solvent B (acetonitrile) were used in the following gradients: initial, 5 mL/100 mL B; 0–15 min, to 10 mL/100 mL B linear; 15–25 min to 60 mL/100 mL B linear; followed by washing and reconditioning of the column. A flow rate of 1 mL/min and an oven temperature of 15 °C with the injection of the filtrate (20 μL) on the HPLC system. The fluorescence detection was recorded at excitation wavelength of 278 nm and emission wavelength of 360 nm. The calibration curves, which were based on peak area, were established using (+)-catechin, (−)-epicatechin, (+)-catechins, and (−)-epicatechin-phloroglucinol adduct standards. The average degree of polymerization was measured by calculating the molar ratio of all the flavan-3-ol units (phloroglucinol adducts + terminal units) to (−)-epicatechin and (+)-catechin, which correspond to terminal units. Quantification of the (+)-catechin, (−)-epicatechin, (+)-catechin, and (−)-epicatechin-phloroglucinol adducts was achieved by using the calibration curves of the corresponding standards (Extrasynthese). All data were obtained in triplicate. The results were expressed as mg per 100 g d.m.

#### 3.3.4. Antioxidant Capacity

For antioxidant activity: the samples (1 g) were mixed with 10 mL of MeOH/water (80:20%, *v*/*v*) and with 1% of HCl, sonicated at 20 °C for 15 min and left for 24 h at 4 °C. Then the extract was again sonicated for 15 min, and centrifuged at 15,000g for 10 min. The ABTS and the FRAP assays were determined according to Re et al. [41] and Benzie and Strain [42], respectively, by Synergy H1 spectrophotometer (BioTek Instruments Inc., Winnoski, VT, USA). The antioxidant capacity was expressed as mmol of Trolox per 100 g d.m.

#### 3.3.5. Statistical Analysis

Statistical analysis, one-way ANOVA and principal component analysis (PCA) were conducted using Statistica version 12.5 (StatSoft, Kraków, Poland). Significant differences (*p* ≤ 0.05) between average values were evaluated by one-way ANOVA and HSD Tukey multiple range test.

## 4. Conclusions

The concentration of carriers had a stronger impact on the content of polyphenolic compounds and antioxidant capacity than the type of carrier or drying method. The choice of a carrier should be done in dependence of the matrix and of the desired composition of bioactive compounds and antioxidant activity. Therefore, maltodextrin can be recommended for powders obtained from the fruit and juice of Saskatoon berry, whereas inulin can be applied in order to produce pomace powders with better functional composition of bioactive compounds. In addition, PCA showed that the freeze-drying process led to the highest content of almost all polyphenolic compounds and their high antioxidant activity, regardless of the matrix tested.

Thus, in future research, properly prepared Saskatoon berry powders with the mentioned carriers will be used as functional additives to design functional food especially foodstuff for enhancing health benefit properties.

## Figures and Tables

**Table 1:** The sum of anthocyanins, phenolic acids, flavan-3-ols, polymeric procyanidins, and total polyphenolic compounds [mg/100 g d.m.] in the fruit, juice, and pomace powders made form Saskatoon berry.

Drying Method	Type of Carriers	Con. [%]	Anthocyanins	Phenolic Acids	Flavonols	Flavavan-3-ols	Polymeric Procyanidins	Total of Polyphenolic Compounds
FRUIT								
FD	Inulin	30	2457.6 ± 662.6 ^1^	1572.4 ± 266.0	365.1 ± 57.5	597.7 ± 493.3	2537.6 ± 493.3	6625.0 ± 1177.7
		40	1887.7 ± 489.4	1175.5 ± 195.0	311.0 ± 46.6	557.5 ± 194.0	1313.9 ± 194.0	4423.7 ± 791.3
		50	1282.0 ± 335.9	932.5 ± 161.6	254.4 ± 35.9	510.6 ± 166.6	1149.0 ± 166.6	3399.4 ± 578.2
	Maltodextrin	30	2195.9 ± 608.3	1505.3 ± 259.5	408.5 ± 64.0	592.8 ± 447.0	2354.3 ± 447.0	6119.6 ± 1073.5
		40	1497.8 ± 382.9	1337.0 ± 230.6	329.4 ± 49.5	560.6 ± 236.3	1490.9 ± 236.3	4375.3 ± 724.0
		50	1111.8 ± 305.1	907.1 ± 147.8	231.0 ± 31.1	463.7 ± 134.6	975.0 ± 134.6	3025.0 ± 505.7
VD/50	Inulin	30	741.3 ± 243.9	1831.3 ± 306.8	342.7 ± 54.4	550.6 ± 381.0	2049.7 ± 381.0	4676.7 ± 959.1
		40	809.5 ± 262.6	1312.9 ± 232.1	271.1 ± 40.0	532.5 ± 261.9	1557.8 ± 261.9	3720.2 ± 687.4
		50	617.5 ± 208.6	980.3 ± 175.5	229.6 ± 31.3	437.7 ± 142.8	973.5 ± 142.8	2602.6 ± 460.4
	Maltodextrin	30	897.8 ± 303.4	1905.5 ± 326.2	359.7 ± 56.5	517.2 ± 269.2	1576.6 ± 269.2	4436.4 ± 841.3
		40	681.7 ± 217.8	1474.7 ± 263.5	270.7 ± 40.2	500.6 ± 177.8	1183.7 ± 177.8	3380.3 ± 645.4
		50	496.6 ± 165.4	963.4 ± 165.6	217.5 ± 29.9	440.5 ± 121.7	885.1 ± 121.7	2375.0 ± 440.6
VD/60	Inulin	30	874.5 ± 288.3	1743.3 ± 288.3	352.2 ± 56.6	575.9 ± 369.3	2029.3 ± 369.3	4703.8 ± 916.3
		40	665.9 ± 215.0	1280.0 ± 208.8	266.0 ± 39.3	497.6 ± 186.1	1218.0 ± 186.1	3203.2 ± 594.7
		50	555.8 ± 189.4	868.5 ± 144.8	222.0 ± 30.5	468.5 ± 210.7	1283.5 ± 210.7	2738.3 ± 540.5
	Maltodextrin	30	735.2 ± 242.1	1708.9 ± 281.9	352.6 ± 56.5	529.3 ± 200.9	1314.4 ± 200.9	3814.9 ± 742.4
		40	592.4 ± 192.4	1200.8 ± 190.7	262.6 ± 39.5	416.0 ± 114.0	835.0 ± 114.0	2667.7 ± 503.9
		50	493.7 ± 164.4	919.0 ± 146.4	226.8 ± 31.3	433.5 ± 84.6	699.4 ± 84.6	2143.5 ± 392.1
POMACE								
FD	Inulin	30	3711.7 ± 928.2	1783.7 ± 286.1	740.3 ± 133.6	532.1 ± 753.6	3481.1 ± 753.6	7873.7 ± 1464.8
		40	3139.3 ± 790.5	1512.9 ± 241.2	636.5 ± 111.8	491.8 ± 626.2	2942.1 ± 626.2	6834.5 ± 1262.3
		50	2287.2 ± 576.1	1173.9 ± 178.0	652.0 ± 126.6	415.9 ± 539.1	2521.5 ± 539.1	7533.7 ± 1611.3
	Maltodextrin	30	3399.8 ± 859.5	1730.9 ± 285.0	820.1 ± 141.1	511.3 ± 495.5	2454.2 ± 495.5	7465.6 ± 1348.2
		40	2475.3 ± 622.3	1361.2 ± 220.8	654.6 ± 109.3	512.9 ± 608.6	2896.2 ± 608.6	6654.0 ± 1259.2
		50	2267.8 ± 570.9	1001.0 ± 154.0	481.8 ± 76.9	408.0 ± 647.5	2937.6 ± 647.5	6390.0 ± 1329.4
VD/50	Inulin	30	2374.5 ± 806.6	1560.8 ± 238.1	565.1 ± 94.6	465.2 ± 637.0	2956.9 ± 637.0	6336.2 ± 1211.3
		40	1723.9 ± 583.8	1302.5 ± 221.5	431.1 ± 69.8	431.3 ± 535.7	2524.8 ± 535.7	5470.4 ± 1040.6
		50	1573.3 ± 533.4	1201.1 ± 214.4	380.4 ± 58.9	412.3 ± 364.5	1835.5 ± 364.5	5669.8 ± 1089.7
	Maltodextrin	30	2574.3 ± 870.2	1582.5 ± 235.2	555.1 ± 90.8	522.1 ± 620.2	2951.2 ± 620.2	6785.3 ± 1247.4
		40	2160.8 ± 733.1	1388.8 ± 215.5	471.1 ± 73.5	471.1 ± 407.2	2066.5 ± 407.2	5093.3 ± 883.4
		50	1564.5 ± 530.7	1243.7 ± 208.2	369.5 ± 56.0	427.4 ± 310.9	1642.3 ± 310.9	4647.2 ± 791.1
VD/60	Inulin	30	1705.3 ± 574.1	1293.1 ± 196.9	495.0 ± 78.3	533.5 ± 528.1	2604.6 ± 528.1	5639.4 ± 1076.5
		40	1663.4 ± 562.1	1138.9 ± 180.3	450.3 ± 68.4	545.7 ± 486.5	2452.5 ± 486.5	4529.9 ± 1013.9
		50	870.2 ± 284.1	1096.7 ± 170.4	321.6 ± 44.3	465.5 ± 421.3	2112.7 ± 421.3	5998.2 ± 1201.2
	Maltodextrin	30	2744.5 ± 924.5	1345.0 ± 190.2	597.1 ± 93.1	548.4 ± 560.0	2743.8 ± 560.0	6186.0 ± 1163.4
		40	2004.1 ± 680.3	1166.4 ± 176.7	475.2 ± 70.4	513.0 ± 449.6	2275.3 ± 449.6	4850.6 ± 932.4
		50	1338.5 ± 451.5	1048.4 ± 168.4	368.4 ± 53.7	456.5 ± 312.0	1674.1 ± 312.0	3008.6 ± 767.9
JUICE								
FD	Inulin	30	232.5 ±60.4	1400.3 ± 215.1	187.2 ± 26.5	449.0 ± 94.9	770.2 ± 94.9	2330.2 ± 644.0
		40	157.7 ±40.7	1077.5 ± 186.9	143.6 ± 17.1	406.5 ± 93.6	715.8 ± 93.6	2008.0 ± 490.3
		50	133.3 ±30.7	867.8 ± 151.0	137.2 ± 14.4	324.2 ± 94.0	630.4 ± 94.0	1671.1 ± 403.8
	Maltodextrin	30	197.6 ±49.8	1418.7 ± 223.1	188.8 ± 26.1	447.5 ± 69.0	622.4 ± 69.0	2206.2 ± 640.4
		40	158.5 ±39.6	1162.6 ± 191.8	155.0 ± 18.9	407.4 ± 62.2	525.9 ± 62.2	1843.6 ± 520.9
		50	139.0 ±34.2	875.4 ± 156.5	129.4 ± 13.3	373.7 ± 59.6	460.9 ± 59.6	1404.4 ± 407.3
VD/50	Inulin	30	136.1 ±46.2	1249.8 ± 186.7	189.5 ± 27.2	428.0 ± 90.2	725.5 ± 90.2	2052.6 ± 595.7
		40	54.9 ± 19.3	1008.2 ± 166.1	157.0 ± 19.9	380.2 ± 85.7	668.0 ± 85.7	1788.4 ± 479.2
		50	50.1 ± 17.7	824.7 ± 136.3	138.7 ± 15.1	387.9 ± 85.2	653.5 ± 85.2	1582.1 ± 408.5
	Maltodextrin	30	92.3 ± 31.6	1263.1 ± 193.4	187.9 ± 27.0	433.2 ± 61.1	556.5 ± 61.1	1915.9 ± 582.3
		40	88.7 ± 29.7	1095.0 ± 176.2	164.1 ± 21.1	403.2 ± 60.0	503.4 ± 60.0	1659.1 ± 510.8
		50	69.4 ± 23.0	844.4 ± 139.9	137.9 ± 15.3	375.9 ± 57.5	443.8 ± 57.5	1325.9 ± 400.3
VD/60	Inulin	30	39.6 ± 12.1	1155.5 ± 158.7	195.8 ± 28.5	483.3 ± 94.1	782.4 ± 94.1	2000.8 ± 570.2
		40	22.4 ± 7.8	914.9 ± 146.5	142.7 ± 16.6	408.3 ± 88.2	684.5 ± 88.2	1710.7 ± 446.0
		50	34.4 ± 11.0	878.7 ± 125.9	170.2 ± 21.5	390.4 ± 89.3	680.6 ± 89.3	1661.0 ± 435.7
	Maltodextrin	30	94.6 ± 31.0	1271.5 ± 172.6	212.7 ± 32.2	456.9 ± 71.4	628.0 ± 71.4	1989.0 ± 593.4
		40	80.2 ± 26.0	1078.1 ± 159.3	176.0 ± 23.0	415.6 ± 64.3	529.1 ± 64.3	1630.2 ± 538.9
		50	57.3 ± 18.7	825.4 ± 125.9	137.9 ± 15.0	377.9 ± 62.8	458.2 ± 62.8	1298.6 ± 413.3

^1^ Values are expressed as the mean (*n* = 3) ± standard deviation. ND, not detected; FD, freeze-drying; VD/50, vacuum-drying at 50 °C; VD/60, vacuum-drying at 60 °C.

**Table 2:** Antioxidant activity [mmol Trolox/100 g d.m.] of the fruit, juice, and pomace powders made from Saskatoon berry.

Drying Method	Type of Carriers	Con. [%]	Antioxidant Capacity [mmol Trolox/100 g d.m.]	
			ABTS	FRAP
FRUIT				
FD	Inulin	30	5.2 ± 0.4 ^1^	3.4 ± 0.1
		40	3.9 ± 0.1	2.5 ± 0.1
		50	3.8 ± 0.2	2.1 ± 0.2
	Maltodextrin	30	5.4 ± 0.1	4.0 ± 0.1
		40	4.4 ± 0.1	3.1 ± 0.3
		50	2.4 ± 0.1	1.9 ± 0.2
VD/50	Inulin	30	4.9 ± 0.1	3.0 ± 0.1
		40	4.2 ± 0.3	2.7 ± 0.1
		50	3.1 ± 0.1	2.0 ± 0.2
	Maltodextrin	30	4.9 ± 0.1	3.4 ± 0.1
		40	3.2 ± 0.1	2.2 ± 0.1
		50	1.9 ± 0.1	1.5 ± 0.1
VD/60	Inulin	30	5.8 ± 0.5	3.9 ± 0.1
		40	3.4 ± 0.1	2.4 ± 0.2
		50	2.7 ± 0.3	2.1 ± 0.2
	Maltodextrin	30	4.9 ± 0.6	3.3 ± 0.3
		40	2.9 ± 0.5	2.3 ± 0.2
		50	2.5 ± 0.1	1.5 ± 0.1
POMACE				
FD	Inulin	30	10.0 ± 0.9	7.3 ± 0.1
		40	9.0 ± 0.2	6.7 ± 0.5
		50	7.9 ± 0.3	4.9 ± 0.2
	Maltodextrin	30	11.6 ± 0.5	7.9 ± 0.2
		40	9.7 ± 0.4	6.9 ± 0.1
		50	6.9 ± 0.2	5.3 ± 0.5
VD/50	Inulin	30	9.6 ± 0.4	6.0 ± 0.4
		40	7.7 ± 0.3	4.8 ± 0.1
		50	5.7 ± 0.1	4.1 ± 0.5
	Maltodextrin	30	8.6 ± 0.1	5.4 ± 0.1
		40	7.7 ± 0.5	4.8 ± 0.2
		50	6.1 ± 0.3	3.8 ± 0.4
VD/60	Inulin	30	7.9 ± 0.2	4.8 ± 0.3
		40	6.3 ± 0.5	4.0 ± 0.1
		50	5.7 ± 0.5	3.7 ± 0.1
	Maltodextrin	30	7.7 ± 0.1	4.7 ± 0.6
		40	6.6 ± 0.2	4.0 ± 0.3
		50	5.3 ± 0.4	3.0 ± 0.1
JUICE				
FD	Inulin	30	1.7 ± 0.1	1.2 ± 0.1
		40	1.1 ± 0.1	0.8 ± 0.1
		50	0.8 ± 0.1	0.6 ± 0.1
	Maltodextrin	30	1.5 ± 0.1	1.1 ± 0.1
		40	0.9 ± 0.2	0.8 ± 0.1
		50	0.7 ± 0.1	0.5 ± 0.1
VD/50	Inulin	30	1.5 ± 0.1	1.2 ± 0.1
		40	1.2 ± 0.1	0.9 ± 0.1
		50	1.0 ± 0.1	0.7 ± 0.1
	Maltodextrin	30	1.4 ± 0.3	1.3 ± 0.1
		40	1.2 ± 0.1	0.8 ± 0.1
		50	0.7 ± 0.1	0.5 ± 0.1
VD/60	Inulin	30	1.6 ± 0.1	1.2 ± 0.1
		40	1.2 ± 0.1	0.7 ± 0.1
		50	0.9 ± 0.1	0.9 ± 0.1
	Maltodextrin	30	1.4 ± 0.2	1.1 ± 0.2
		40	1.1 ± 0.3	0.9 ± 0.1
		50	0.7 ± 0.2	0.5 ± 0.1

^1^ Values are expressed as the mean (*n* = 3) ± standard deviation. ND, not detected; FD, freeze-drying; VD/50, vacuum-drying at 50 °C; VD/60, vacuum-drying at 60 °C.

## Supplementary Materials

The following are available online, Table S1. Identification and quantification of flavan-3-ols [mg/100 g d.m.] in fruit, juice, and pomace powders made form Saskatoon berry, Table S2. Identification and quantification of anthocyanins [mg/100 g d.m.] in the fruit, juice, and pomace powders made form Saskatoon berry, Table S3. Identification and quantification of phenolic acids [mg/100 g d.m.] in the fruit, juice, and pomace powders made form Saskatoon berry, Table S4. Identification and quantification of flavonols [mg/100 g d.m.] in the fruit, juice, and pomace powders made form Saskatoon berry.

## References

[1] Lavola A., Karjalainen R., Julkunen-Tiitto R. (2012). Bioactive Polyphenols in Leaves, Stems, and Berries of Saskatoon (*Amelanchier alnifolia* Nutt.) Cultivars. J. Agric. Food Chem..

[2] Bakowska-Barczak A.M., Kolodziejczyk P. (2008). Evaluation of Saskatoon Berry (*Amelanchier alnifolia* Nutt.) Cultivars for their Polyphenol Content, Antioxidant Properties, and Storage Stability. J. Agric. Food Chem..

[3] Jurikova T., Balla S., Sochor J., Pohanka M., Mlcek J., Baron M. (2013). Flavonoid Profile of Saskatoon Berries (*Amelanchier alnifolia* Nutt.) and Their Health Promoting Effects. Molecules.

[4] Lachowicz S., Oszmiański J., Pluta S. (2017). The composition of bioactive compounds and antioxidant activity of Saskatoon berry (*Amelanchier alnifolia* Nutt.) genotypes grown in central Poland. Food Chem..

[5] Rodríguez-Roque M.J., Rojas-Graü M.A., Elez-Martinez P., Martin-Belloso O. (2013). Soymilk phenolic compounds, isoflavones and antioxidant activity as affected by in vitro gastrointestinal digestion. Food Chem..

[6] Lachowicz S., Seliga Ł., Pluta S. (2020). Distribution of phytochemicals and antioxidative potency in fruit peel, flesh, and seeds of Saskatoon berry. Food Chem..

[7] Ioannou I., Hafsa I., Hamdi S., Charbonnel C., Ghoul M. (2012). Review of the effects of food processing and formulation on flavonol and anthocyanin behaviour. J. Food Eng..

[8] Augustin M.A., Hemar Y. (2009). Nano- and micro-structured assemblies for encapsulation of food ingredients. Chem. Soc. Rev..

[9] Fang Z., Bhandari B. (2011). Effect of spray drying and storage on the stability of bayberry polyphenols. Food Chem..

[10] Kha T., Nguyen M.H., Roach P. (2010). Effects of spray drying conditions on the physicochemical and antioxidant properties of the Gac (*Momordica cochinchinensis*) fruit aril powder. J. Food Eng..

[11] Wang Y., Lu Z., Lv F., Bie X. (2009). Study on microencapsulation of curcumin pigments by spray drying. Eur. Food Res. Technol..

[12] Saikia S., Mahnot N.K., Mahanta C.L. (2015). Optimisation of phenolic extraction from Averrhoa carambola pomace by response surface methodology and its microencapsulation by spray and freeze drying. Food Chem..

[13] Shoaib M., Shehzad A., Omar M., Rakha A., Raza H., Sharif H.R., Shakeel A., Ansari A., Niazi S. (2016). Inulin: Properties, health benefits and food applications. Carbohydr. Polym..

[14] Silva P.I., Stringheta P.C., Teofilo R., Oliveira I. (2013). Parameter optimization for spray-drying microencapsulation of jaboticaba (*Myrciaria jaboticaba*) peel extracts using simultaneous analysis of responses. J. Food Eng..

[15] Michalska-Ciechanowska A., Wojdyło A., Lech K., Łysiak G., Figiel A. (2016). Physicochemical properties of whole fruit plum powders obtained using different drying technologies. Food Chem..

[16] Michalska-Ciechanowska A., Wojdyło A., Brzezowska J., Majerska J., Ciska E. (2019). The Influence of Inulin on the Retention of Polyphenolic Compounds during the Drying of Blackcurrant Juice. Molecules.

[17] Lachowicz S., Michalska A., Lech K., Majerska J., Oszmiański J., Figiel A. (2019). Comparison of the effect of four drying methods on polyphenols in saskatoon berry. LWT.

[18] De Souza D.R., Willems J.L., Low N.H. (2019). Phenolic composition and antioxidant activities of saskatoon berry fruit and pomace. Food Chem..

[19] Slimestad R., Torskangerpoll K., Nateland H.S., Johannessen T., Giske N.H. (2005). Flavonoids from black chokeberries, Aronia melanocarpa. J. Food Compos. Anal..

[20] White B.L., Howard L.R., Prior R.L. (2010). Proximate and Polyphenolic Characterization of Cranberry Pomace†. J. Agric. Food Chem..

[21] Oszmiański J., Lachowicz S. (2016). Effect of the Production of Dried Fruits and Juice from Chokeberry (*Aronia melanocarpa* L.) on the Content and Antioxidative Activity of Bioactive Compounds. Molecules.

[22] Gouw V.P., Jung J., Zhao Y. (2017). Functional properties, bioactive compounds, and in vitro gastrointestinal digestion study of dried fruit pomace powders as functional food ingredients. LWT.

[23] Jurikova T., Sochor J., Rop O., Mlcek J., Balla S., Szekeres L., Žitný R., Zitka O., Adam V., Kizek R. (2012). Evaluation of Polyphenolic Profile and Nutritional Value of Non-Traditional Fruit Species in the Czech Republic — A Comparative Study. Molecules.

[24] Lachowicz S., Oszmiański J., Seliga Ł., Pluta S. (2017). Phytochemical Composition and Antioxidant Capacity of Seven Saskatoon Berry (*Amelanchier alnifolia* Nutt.) Genotypes Grown in Poland. Molecules.

[25] Mazza G., Cottrell T. (2008). Carotenoids and cyanogenic glucosides in saskatoon berries (*Amelanchier alnifolia* Nutt.). J. Food Compos. Anal..

[26] Li W., Hydamaka A.W., Lowry L., Beta T. (2009). Comparison of antioxidant capacity and phenolic compounds of berries, chokecherry and seabuckthorn. Open Life Sci..

[27] Fang J., Huang J. (2019). Accumulation of plasma levels of anthocyanins following multiple saskatoon berry supplements. Xenobiotica.

[28] Zhao R., Khafipour E., Sepehri S., Huang F., Beta T., Shen G.X. (2019). Impact of Saskatoon berry powder on insulin resistance and relationship with intestinal microbiota in high fat-high sucrose diet-induced obese mice. J. Nutr. Biochem..

[29] Bakowska-Barczak A.M., Kolodziejczyk P.P. (2011). Black currant polyphenols: Their storage stability and microencapsulation. Ind. Crop. Prod..

[30] Sáenz C., Tapia S., Chávez J., Robert P. (2009). Microencapsulation by spray drying of bioactive compounds from cactus pear (*Opuntia ficus-indica*). Food Chem..

[31] Michalska-Ciechanowska A., Wojdyło A., Honke J., Ciska E., Andlauer W. (2018). Drying-induced physico-chemical changes in cranberry products. Food Chem..

[32] Daza L.D., Fujita A., Granato D., Favaro-Trindade C.S., Genovese M.I. (2017). Functional properties of encapsulated Cagaita (*Eugenia dysenterica* DC.) fruit extract. Food Biosci..

[33] Michalska-Ciechanowska A., Wojdyło A., Łysiak G., Figiel A. (2017). Chemical Composition and Antioxidant Properties of Powders Obtained from Different Plum Juice Formulations. Int. J. Mol. Sci..

[34] White B.L., Howard L.R., Prior R.L. (2011). Impact of Different Stages of Juice Processing on the Anthocyanin, Flavonol, and Procyanidin Contents of Cranberries. J. Agric. Food Chem..

[35] Michalska-Ciechanowska A., Wojdyło A., Łysiak G., Lech K., Figiel A. (2017). Functional relationships between phytochemicals and drying conditions during the processing of blackcurrant pomace into powders. Adv. Powder Technol..

[36] Cai Y., Corke H. (2000). Production and Properties of Spray-dried Amaranthus Betacyanin Pigments. J. Food Sci..

[37] Patras A., Brunton N., O’Donnell C.P., Tiwari B. (2010). Effect of thermal processing on anthocyanin stability in foods; mechanisms and kinetics of degradation. Trends Food Sci. Technol..

[38] Tiwari U., Cummins E. (2013). Factors influencing levels of phytochemicals in selected fruit and vegetables during pre- and post-harvest food processing operations. Food Res. Int..

[39] Piga A., Del Caro A., Corda G. (2003). From Plums to Prunes: Influence of Drying Parameters on Polyphenols and Antioxidant Activity. J. Agric. Food Chem..

[40] Siddiq M., Dolan K. (2017). Characterization of polyphenol oxidase from blueberry (*Vaccinium corymbosum* L.). Food Chem..

[41] Re R., Pellegrini N., Proteggente A., Pannala A., Yang M., Rice-Evans C. (1999). Antioxidant activity applying an improved ABTS radical cation decolorization assay. Free. Radic. Boil. Med..

[42] Benzie I., Strain J. (1996). The Ferric Reducing Ability of Plasma (FRAP) as a Measure of “Antioxidant Power”: The FRAP Assay. Anal. Biochem..

