# Supplementary material for: The Impact of Maltodextrin and Inulin on the Protection of Natural Antioxidants in Powders Made of Saskatoon Berry Fruit, Juice, and Pomace as Functional Food Ingredients
Source: Molecules. 2020 Apr 15;25(8):1805. doi: 10.3390/molecules25081805 (PMC7221788; doi:10.3390/molecules25081805)
Supplement: Supplementary file 1 [file molecules-25-01805-s001.pdf]

molecules

MDPI

Article

The impact of maltodextrin and inulin on the protection of natural antioxidants in powders made of Saskatoon berry fruit, juice, and pomace as functional food ingredients

Sabina Lachowicz [1,*], Anna Michalska-Ciechanowska [2] and Jan Oszmiański [2]

[1] Department of Fermentation and Cereals Technology, Faculty of Biotechnology and Food Science, Wrocław University of Environmental and Life Sciences, 51–630 Wrocław, Poland
[2] Department of Fruit, Vegetable and Plant Nutraceutical Technology, Faculty of Biotechnology and Food Science, Wrocław University of Environmental and Life Sciences, 51–630 Wrocław, Poland; anna.michalska@upwr.edu.pl (A.M.-C.), jan.oszmianski@upwr.edu.pl (J.O.)
* Correspondence: sabina.lachowicz@upwr.edu.pl

Table 1. Identification and quantification of flavan-3-ols [mg/100 g d.m.] in fruit, juice, and pomace powders made form Saskatoon berry.

Drying method	Carrier	Con. [%]	PB4	PB4	PA2	PB2	Cat	PB3	Epi	PB3	PB4	PB4	PB3	PB3	PB4	PB4	DP
								FRUIT									
FD	Inulin	30	237.8 ± 4.8	7.7 ± 0.2	82.7 ± 1.7	49.5 ± 1.0	31.6 ± 0.6	42.4 ± 0.8	4.4 ± 0.1	ND	34.3 ± 0.7	5.0 ± 0.1	16.4 ± 0.3	6.5 ± 0.1	30.5 ± 0.6	49.1 ± 1.0	8.6 ± 0.2
		40	235.7 ± 4.7	4.0 ± 0.1	76.3 ± 1.5	47.2 ± 0.9	22.8 ± 0.5	33.3 ± 0.7	5.2 ± 0.1	ND	26.6 ± 0.5	5.2 ± 0.1	14.3 ± 0.3	6.0 ± 0.1	32.5 ± 0.7	48.4 ± 1.0	9.7 ± 0.2
		50	245.1 ± 4.9	3.1 ± 0.1	50.0 ± 1.0	45.5 ± 0.9	15.1 ± 0.3	25.6 ± 0.5	6.6 ± 0.1	ND	25.0 ± 0.5	5.3 ± 0.1	13.6 ± 0.3	4.1 ± 0.1	32.8 ± 0.7	38.9 ± 0.8	7.6 ± 0.2
	Maltodextrin	30	212.9 ± 4.3	4.8 ± 0.1	70.5 ± 1.4	53.1 ± 1.1	24.8 ± 0.5	58.1 ± 1.2	5.6 ± 0.1	ND	58.7 ± 1.2	4.9 ± 0.1	16.5 ± 0.3	5.9 ± 0.1	30.9 ± 0.6	46.0 ± 0.9	8.1 ± 0.2
		40	220.3 ± 4.4	3.5 ± 0.1	79.6 ± 1.6	38.6 ± 0.8	24.6 ± 0.5	39.8 ± 0.8	2.1 ± 0.0	ND	52.4 ± 1.0	4.8 ± 0.1	15.6 ± 0.3	5.8 ± 0.1	29.3 ± 0.6	44.4 ± 0.9	9.7 ± 0.2
		50	218.2 ± 4.4	2.3 ± 0.1	48.2 ± 1.0	25.7 ± 0.5	15.2 ± 0.3	27.4 ± 0.5	2.7 ± 0.1	ND	27.2 ± 0.5	4.2 ± 0.1	14.1 ± 0.3	3.8 ± 0.1	34.0 ± 0.7	40.8 ± 0.8	7.7 ± 0.2
VD/50	Inulin	30	229.4 ± 4.6	3.4 ± 0.1	65.3 ± 1.3	32.8 ± 0.7	22.4 ± 0.4	40.0 ± 0.8	2.7 ± 0.1	3.0 ± 0.1	30.3 ± 0.6	7.8 ± 0.2	29.9 ± 0.6	5.1 ± 0.1	32.4 ± 0.6	46.1 ± 0.9	8.6 ± 0.2
		40	244.5 ± 4.9	2.5 ± 0.1	45.2 ± 0.9	51.9 ± 1.0	17.4 ± 0.3	24.5 ± 0.5	2.6 ± 0.1	2.7 ± 0.1	38.6 ± 0.8	5.8 ± 0.1	11.8 ± 0.2	4.9 ± 0.1	33.6 ± 0.7	46.6 ± 0.9	9.5 ± 0.2
		50	237.3 ± 4.7	2.1 ± 0.1	31.1 ± 0.6	15.5 ± 0.3	11.5 ± 0.2	20.8 ± 0.4	2.1 ± 0.1	0.7 ± 0.0	28.0 ± 0.6	5.5 ± 0.1	9.3 ± 0.2	3.5 ± 0.1	29.4 ± 0.6	41.3 ± 0.8	8.4 ± 0.2
	Maltodextrin	30	218.5 ± 4.4	3.7 ± 0.1	51.2 ± 1.0	30.1 ± 0.6	20.6 ± 0.4	43.9 ± 0.9	3.6 ± 0.1	5.8 ± 0.1	31.4 ± 0.6	6.8 ± 0.1	22.1 ± 0.4	4.0 ± 0.1	29.6 ± 0.6	46.0 ± 0.9	8.8 ± 0.2
		40	246.7 ± 4.9	2.4 ± 0.1	45.5 ± 0.9	16.5 ± 0.3	16.5 ± 0.3	31.3 ± 0.6	2.7 ± 0.1	3.7 ± 0.1	30.6 ± 0.6	5.6 ± 0.1	16.4 ± 0.3	2.3 ± 0.1	30.2 ± 0.6	50.2 ± 1.0	9.5 ± 0.2
		50	237.4 ± 4.7	2.1 ± 0.1	33.0 ± 0.7	12.6 ± 0.3	9.9 ± 0.2	22.4 ± 0.4	2.2 ± 0.1	1.2 ± 0.0	27.8 ± 0.6	3.8 ± 0.1	14.5 ± 0.3	1.9 ± 0.1	28.6 ± 0.6	43.2 ± 0.9	7.9 ± 0.2
VD/60	Inulin	30	236.0 ± 4.7	2.8 ± 0.1	68.0 ± 1.4	36.3 ± 0.7	22.4 ± 0.4	49.8 ± 1.0	2.9 ± 0.1	5.0 ± 0.1	55.0 ± 1.1	11.0 ± 0.2	3.6 ± 0.1	3.7 ± 0.1	33.7 ± 0.7	45.9 ± 0.9	8.9 ± 0.2
		40	238.3 ± 4.8	2.5 ± 0.1	44.3 ± 0.9	25.3 ± 0.5	13.0 ± 0.3	29.8 ± 0.6	2.6 ± 0.1	2.8 ± 0.1	37.6 ± 0.8	10.6 ± 0.2	12.9 ± 0.3	3.2 ± 0.1	30.8 ± 0.6	44.0 ± 0.9	9.7 ± 0.2
		50	246.1 ± 4.9	2.2 ± 0.1	40.2 ± 0.8	27.0 ± 0.5	11.6 ± 0.2	21.9 ± 0.4	1.6 ± 0.1	1.9 ± 0.1	29.9 ± 0.6	3.2 ± 0.1	7.9 ± 0.2	3.3 ± 0.1	29.7 ± 0.6	41.9 ± 0.8	8.4 ± 0.2
	Maltodextrin	30	229.6 ± 4.6	3.3 ± 0.1	49.1 ± 1.0	23.1 ± 0.5	16.5 ± 0.3	43.7 ± 0.9	3.6 ± 0.1	6.1 ± 0.1	52.3 ± 1.0	7.2 ± 0.1	20.2 ± 0.4	1.7 ± 0.1	28.7 ± 0.6	44.3 ± 0.9	8.6 ± 0.2
		40	219.5 ± 4.4	3.0 ± 0.1	37.8 ± 0.8	18.0 ± 0.4	11.1 ± 0.2	26.3 ± 0.5	2.6 ± 0.1	4.2 ± 0.1	17.1 ± 0.3	4.2 ± 0.1	19.8 ± 0.4	1.0 ± 0.1	17.9 ± 0.4	33.3 ± 0.7	9.7 ± 0.2
		50	237.8 ± 4.8	2.2 ± 0.1	27.4 ± 0.5	7.3 ± 0.1	8.4 ± 0.2	20.3 ± 0.4	2.3 ± 0.1	2.4 ± 0.1	28.6 ± 0.6	3.1 ± 0.1	14.8 ± 0.3	1.0 ± 0.1	31.5 ± 0.6	46.4 ± 0.9	8.4 ± 0.2
								POMACE									
FD	Inulin	30	223.4 ± 4.5	7.5 ± 0.2	7.5 ± 0.1	14.8 ± 0.3	3.9 ± 0.1	66.2 ± 1.3	14.2 ± 0.3	9.8 ± 0.2	27.3 ± 0.5	15.5 ± 0.3	63.5 ± 1.3	6.6 ± 0.1	30.1 ± 0.6	41.8 ± 0.8	9.6 ± 0.2
		40	231.3 ± 4.6	6.9 ± 0.1	3.6 ± 0.1	13.7 ± 0.3	3.6 ± 0.1	45.8 ± 0.9	12.3 ± 0.2	8.8 ± 0.2	25.4 ± 0.5	12.2 ± 0.2	51.4 ± 1	3.7 ± 0.1	32.1 ± 0.6	40.9 ± 0.8	9.7 ± 0.2

		50	230.8 ± 4.6	5.1 ± 0.1	3.0 ± 0.1	8.9 ± 0.2	2.8 ± 0.1	41.1 ± 0.8	11.8 ± 0.2	7.7 ± 0.2	13.1 ± 0.1	7.4 ± 0.1	22.5 ± 0.4	5.1 ± 0.1	26.6 ± 0.5	40.0 ± 0.8	9.5 ± 0.2
	Maltodextrin	30	234.4 ± 4.7	7.5 ± 0.1	4.6 ± 0.1	12.5 ± 0.2	5.4 ± 0.1	65.0 ± 1.3	15.0 ± 0.3	10.5 ± 0.2	32.2 ± 0.6	11.8 ± 0.2	31.2 ± 0.6	12.2 ± 0.2	27.3 ± 0.5	41.9 ± 0.8	9.0 ± 0.2
		40	229.9 ± 4.6	7.2 ± 0.1	4.6 ± 0.1	11.0 ± 0.2	3.9 ± 0.1	56.9 ± 1.1	13.1 ± 0.3	9.8 ± 0.2	26.5 ± 0.5	12.4 ± 0.2	65.6 ± 1.3	7.1 ± 0.1	26.8 ± 0.5	38.0 ± 0.8	9.7 ± 0.2
		50	199.9 ± 4.0	5.7 ± 0.1	3.3 ± 0.1	10.2 ± 0.2	2.2 ± 0.1	44.1 ± 0.9	12.2 ± 0.2	5.4 ± 0.1	16.1 ± 0.3	8.5 ± 0.2	28.9 ± 0.6	7.4 ± 0.1	24.5 ± 0.5	39.7 ± 0.8	9.9 ± 0.2
VD/50	Inulin	30	221.4 ± 4.4	3.8 ± 0.1	4.2 ± 0.1	18.2 ± 0.4	16.6 ± 0.3	41.0 ± 0.8	29.9 ± 0.6	5.9 ± 0.1	20.8 ± 0.4	10.1 ± 0.2	20 ± 0.4	10.1 ± 0.2	26.3 ± 0.5	37.1 ± 0.7	9.3 ± 0.2
		40	234.2 ± 4.7	3.1 ± 0.1	4.0 ± 0.1	14.1 ± 0.3	10.0 ± 0.2	28.8 ± 0.6	21.6 ± 0.4	2.7 ± 0.1	16.8 ± 0.3	8.5 ± 0.2	16.0 ± 0.3	6.5 ± 0.1	23.1 ± 0.5	42.0 ± 0.8	10.0 ± 0.2
		50	233.3 ± 4.7	2.6 ± 0.1	1.9 ± 0.0	11.3 ± 0.2	6.6 ± 0.1	22.1 ± 0.4	18.6 ± 0.4	3.6 ± 0.1	14.0 ± 0.3	4.6 ± 0.1	14.6 ± 0.3	6.8 ± 0.1	30.2 ± 0.6	42.0 ± 0.8	9.8 ± 0.2
	Maltodextrin	30	237.3 ± 4.7	4.9 ± 0.1	5.0 ± 0.1	18.5 ± 0.4	12.5 ± 0.3	43.1 ± 0.9	27.3 ± 0.5	5.2 ± 0.1	24.7 ± 0.5	8.1 ± 0.2	52.2 ± 1.0	13.1 ± 0.3	30.2 ± 0.6	40.2 ± 0.8	10.0 ± 0.2
		40	232.2 ± 4.6	4.6 ± 0.1	3.6 ± 0.1	14.3 ± 0.3	11.2 ± 0.2	30.3 ± 0.6	22.2 ± 0.4	5.3 ± 0.1	17.0 ± 0.3	6.3 ± 0.1	42.2 ± 0.8	10.5 ± 0.2	28.6 ± 0.6	42.9 ± 0.9	10.2 ± 0.2
		50	229.1 ± 4.6	3.4 ± 0.1	2.5 ± 0.1	11.6 ± 0.2	6.6 ± 0.1	31.5 ± 0.6	17.2 ± 0.3	3.8 ± 0.1	14.1 ± 0.3	5.7 ± 0.1	27.1 ± 0.5	7.6 ± 0.2	28.2 ± 0.6	39.0 ± 0.8	10.1 ± 0.2
VD/60	Inulin	30	235.9 ± 4.7	2.0 ± 0.1	4.6 ± 0.1	71.6 ± 1.4	14.7 ± 0.3	33.9 ± 0.7	31.1 ± 0.6	2.7 ± 0.1	19.2 ± 0.4	10.5 ± 0.2	26.4 ± 0.5	8.4 ± 0.2	29.5 ± 0.6	43.1 ± 0.9	10.7 ± 0.2
		40	260.9 ± 5.2	2.2 ± 0.1	1.6 ± 0.1	71.0 ± 1.4	13.1 ± 0.3	32.3 ± 0.6	27.7 ± 0.6	2.4 ± 0.1	17.0 ± 0.3	9.0 ± 0.2	21.1 ± 0.4	9.8 ± 0.2	30.8 ± 0.6	46.9 ± 0.9	11.0 ± 0.2
		50	252.1 ± 5.0	0.7 ± 0.1	7.5 ± 0.2	33.4 ± 0.7	10.4 ± 0.2	26.3 ± 0.5	21.2 ± 0.4	3.2 ± 0.1	14.5 ± 0.3	3.7 ± 0.1	15.5 ± 0.3	7.7 ± 0.2	24.3 ± 0.5	45.2 ± 0.9	9.8 ± 0.2
	Maltodextrin	30	250.1 ± 5.0	6.5 ± 0.1	8.2 ± 0.2	28.1 ± 0.6	16.9 ± 0.3	53.8 ± 1.1	30.3 ± 0.6	2.9 ± 0.1	21.2 ± 0.4	11.0 ± 0.2	31.6 ± 0.6	10.8 ± 0.2	30.5 ± 0.6	46.4 ± 0.9	10.8 ± 0.2
		40	248.5 ± 5.0	4.1 ± 0.1	7.0 ± 0.1	23.9 ± 0.5	13.7 ± 0.3	45.0 ± 0.9	25.7 ± 0.5	3.3 ± 0.1	16.1 ± 0.3	8.0 ± 0.2	31.1 ± 0.6	8.9 ± 0.2	31.7 ± 0.6	46.2 ± 0.9	10.7 ± 0.2
		50	248.1 ± 5.0	3.7 ± 0.1	1.7 ± 0.1	16.3 ± 0.3	9.1 ± 0.2	26.5 ± 0.5	24.7 ± 0.5	5.2 ± 0.1	11.2 ± 0.2	5.9 ± 0.1	17.1 ± 0.3	7.7 ± 0.2	34.3 ± 0.7	45.0 ± 0.9	10.5 ± 0.2
JUICE																	
FD	Inulin	30	237.6 ± 4.8	6.8 ± 0.1	8.7 ± 0.2	19.4 ± 0.4	18.8 ± 0.4	38.5 ± 0.8	2.4 ± 0.1	1.0 ± 0.1	29.5 ± 0.6	6.0 ± 0.1	5.1 ± 0.1	2.1 ± 0.1	28.8 ± 0.6	44.3 ± 0.9	8.7 ± 0.2
		40	238.8 ± 4.8	4.5 ± 0.1	7.9 ± 0.2	14.9 ± 0.3	14.5 ± 0.3	22.8 ± 0.5	1.4 ± 0.1	0.7 ± 0.1	19.0 ± 0.4	4.3 ± 0.1	3.4 ± 0.1	1.2 ± 0.1	30.6 ± 0.6	42.6 ± 0.9	8.1 ± 0.2
		50	235.1 ± 4.7	1.5 ± 0.1	6.7 ± 0.1	9.0 ± 0.2	10.4 ± 0.2	16.5 ± 0.3	0.2 ± 0.1	0.6 ± 0.1	4.1 ± 0.1	4.2 ± 0.1	1.3 ± 0.1	2.4 ± 0.1	18.9 ± 0.4	13.4 ± 0.3	8.9 ± 0.2
	Maltodextrin	30	235.8 ± 4.7	6.1 ± 0.1	6.6 ± 0.1	14.2 ± 0.3	15.0 ± 0.3	45.4 ± 0.9	1.9 ± 0.1	0.4 ± 0.1	29.0 ± 0.6	6.6 ± 0.1	7.9 ± 0.2	6.4 ± 0.1	27.8 ± 0.6	44.3 ± 0.9	4.7 ± 0.1
		40	231.7 ± 4.6	4.5 ± 0.1	4.9 ± 0.1	10.3 ± 0.2	8.3 ± 0.2	34.6 ± 0.7	1.4 ± 0.1	0.3 ± 0.1	20.5 ± 0.4	5.9 ± 0.1	6.0 ± 0.1	3.4 ± 0.1	32.7 ± 0.7	42.9 ± 0.9	4.6 ± 0.1
		50	229.9 ± 4.6	3.0 ± 0.1	2.7 ± 0.1	9.8 ± 0.2	7.6 ± 0.2	20.4 ± 0.4	1.0 ± 0.1	0.1 ± 0.1	14.9 ± 0.3	5.5 ± 0.1	4.7 ± 0.1	3.4 ± 0.1	28.2 ± 0.6	42.6 ± 0.9	5.0 ± 0.1
VD/50	Inulin	30	236.3 ± 4.7	4.8 ± 0.1	7.2 ± 0.1	15.4 ± 0.3	14.4 ± 0.3	28.1 ± 0.6	2.8 ± 0.1	6.0 ± 0.1	22.7 ± 0.5	6.8 ± 0.1	5.4 ± 0.1	3.7 ± 0.1	32.6 ± 0.7	41.7 ± 0.8	7.4 ± 0.1
		40	215.2 ± 4.3	3.9 ± 0.1	6.5 ± 0.1	12.3 ± 0.2	12.3 ± 0.2	22.7 ± 0.5	1.8 ± 0.1	5.6 ± 0.1	18.0 ± 0.4	6.3 ± 0.1	5.2 ± 0.1	6.0 ± 0.1	27.9 ± 0.6	36.6 ± 0.7	7.7 ± 0.2
		50	237.1 ± 4.7	2.7 ± 0.1	5.4 ± 0.1	8.9 ± 0.2	10.5 ± 0.2	18.1 ± 0.4	0.8 ± 0.1	3.2 ± 0.1	13.6 ± 0.3	5.8 ± 0.1	4.1 ± 0.1	8.4 ± 0.2	28.4 ± 0.6	41.1 ± 0.8	7.7 ± 0.2
	Maltodextrin	30	229.1 ± 4.6	4.9 ± 0.1	5.5 ± 0.1	16.5 ± 0.3	17.1 ± 0.3	36.0 ± 0.7	2.4 ± 0.1	7.1 ± 0.1	23.7 ± 0.5	6.1 ± 0.1	7.7 ± 0.2	7.6 ± 0.2	27.2 ± 0.5	42.3 ± 0.8	4.6 ± 0.1
		40	229.8 ± 4.6	3.8 ± 0.1	4.1 ± 0.1	11.1 ± 0.2	13.7 ± 0.3	29.2 ± 0.6	1.2 ± 0.1	1.1 ± 0.1	17.7 ± 0.4	4.3 ± 0.1	6.2 ± 0.1	7.6 ± 0.2	30.3 ± 0.6	43.0 ± 0.9	4.6 ± 0.1
		50	226.4 ± 4.5	2.7 ± 0.1	3.3 ± 0.1	8.8 ± 0.2	11.3 ± 0.2	21.6 ± 0.4	1.3 ± 0.1	1.1 ± 0.1	13.2 ± 0.3	3.7 ± 0.1	4.7 ± 0.1	6.7 ± 0.1	30.2 ± 0.6	40.8 ± 0.8	5.0 ± 0.1
VD/60	Inulin	30	263.1 ± 5.3	5.4 ± 0.1	5.9 ± 0.1	19.9 ± 0.4	17.6 ± 0.4	29.7 ± 0.6	5.8 ± 0.1	6.8 ± 0.1	25.5 ± 0.5	7.1 ± 0.1	8.1 ± 0.2	8.0 ± 0.2	31.7 ± 0.6	48.9 ± 1.0	8.5 ± 0.2
		40	244.9 ± 4.9	3.0 ± 0.1	5.9 ± 0.1	14.7 ± 0.3	12.1 ± 0.2	15.3 ± 0.3	3.5 ± 0.1	2.8 ± 0.1	14.7 ± 0.3	4.1 ± 0.1	4.0 ± 0.1	7.4 ± 0.1	30.0 ± 0.6	45.9 ± 0.9	8.5 ± 0.2
		50	233.7 ± 4.7	4.3 ± 0.1	5.5 ± 0.1	11.7 ± 0.2	12.1 ± 0.2	15.2 ± 0.3	3.2 ± 0.1	1.0 ± 0.1	20.1 ± 0.4	3.2 ± 0.1	5.7 ± 0.1	5.7 ± 0.1	26.6 ± 0.5	42.4 ± 0.8	8.8 ± 0.2
	Maltodextrin	30	251.2 ± 5.0	5.9 ± 0.1	6.7 ± 0.1	19.7 ± 0.4	19.4 ± 0.4	33.3 ± 0.7	5.2 ± 0.1	3.7 ± 0.1	26.8 ± 0.5	5.5 ± 0.1	6.8 ± 0.1	5.3 ± 0.1	23.0 ± 0.5	44.5 ± 0.9	4.1 ± 0.1
		40	244.0 ± 4.9	4.5 ± 0.1	4.7 ± 0.1	13.4 ± 0.3	13.8 ± 0.3	29.7 ± 0.6	3.1 ± 0.1	3.0 ± 0.1	18.9 ± 0.4	3.7 ± 0.1	6.3 ± 0.1	5.0 ± 0.1	21.0 ± 0.4	44.5 ± 0.9	4.9 ± 0.1
		50	244.7 ± 4.9	2.8 ± 0.1	3.0 ± 0.1	8.6 ± 0.2	6.9 ± 0.1	20.3 ± 0.4	2.7 ± 0.1	2.8 ± 0.1	13.6 ± 0.3	3.3 ± 0.1	3.8 ± 0.1	4.7 ± 0.1	20.0 ± 0.4	40.7 ± 0.8	5.8 ± 0.1

[1] Values are expressed as the mean (n = 3) ± standard deviation. ND, no detect; FD. freeze-drying; VD/50. vacuum-drying in 50 °C; VD/60. vacuum-drying in 60 °C; PP. polymeric procyanidins; F3O. sum of flavan-3-ols (monomers. oligomers. and polymers); DP. degree of polymerization; Epi. (−)-epicatechin; Cat. (+)-catechin; PB4. B-type procyanidin tetramer; PB3. B-type procyanidin trimer; PB2. B-type procyanidin dimer; PA2. A-type procyanidin dimer.

Table 2. Identification and quantification of anthocyanins [mg/100 g d.m.] in the fruit, juice, and pomace powders made form Saskatoon berry.

Drying method	Type of carriers	Con. [%]	C-3-O-gal	C-3-O-glu	C-3-O-ara	C-3-O-xyl	C	C
				FRUIT				
FD	Inulin	30	1717.5 ± 34.4	460.7 ± 9.2	110.3 ± 2.2	166.3 ± 3.3	2.8 ± 0.1	ND
		40	1276.4 ± 25.5	362.0 ± 7.2	103.0 ± 2.1	144.6 ± 2.9	1.7 ± 0.1	ND
		50	871.3 ± 17.4	258.7 ± 5.2	59.9 ± 1.2	91.0 ± 1.8	1.0 ± 0.1	ND
	Maltodextrin	30	1562.0 ± 31.2	440.0 ± 8.8	66.7 ± 1.3	125.1 ± 2.5	2.1 ± 0.1	ND
		40	1003.4 ± 20.1	277.4 ± 5.5	107.7 ± 2.2	107.4 ± 2.1	1.9 ± 0.1	ND
		50	791.2 ± 15.8	192.5 ± 3.8	52.6 ± 1.1	74.3 ± 1.5	1.2 ± 0.1	ND
VD/50	Inulin	30	619.1 ± 12.4	63.8 ± 1.3	24.7 ± 0.5	31.3 ± 0.6	1.1 ± 0.1	1.4 ± 0.1
		40	668.9 ± 13.4	60.3 ± 1.2	38.5 ± 0.8	38.4 ± 0.8	1.9 ± 0.1	1.5 ± 0.1
		50	527.5 ± 10.5	41.8 ± 0.8	19.5 ± 0.4	26.5 ± 0.5	1.2 ± 0.1	1.0 ± 0.1
	Maltodextrin	30	767.1 ± 15.3	63.9 ± 1.3	20.6 ± 0.4	42.0 ± 0.8	3 ± 0.1.1	1.2 ± 0.1
		40	556.3 ± 11.1	51.9 ± 1.0	38.5 ± 0.8	31.3 ± 0.6	2.4 ± 0.1	1.3 ± 0.1
		50	419.2 ± 8.4	38.3 ± 0.8	15.1 ± 0.3	21.7 ± 0.4	1.1 ± 0.1	1.1 ± 0.1
VD/60	Inulin	30	731.3 ± 14.6	78.4 ± 1.6	23.0 ± 0.5	38.9 ± 0.8	1.5 ± 0.1	1.3 ± 0.1
		40	548.1 ± 11.0	47.7 ± 1.0	38.4 ± 0.8	28.0 ± 0.6	2.3 ± 0.1	1.4 ± 0.1
		50	478.5 ± 9.6	33.7 ± 0.7	20.1 ± 0.4	21.4 ± 0.4	0.7 ± 0.1	1.5 ± 0.1
	Maltodextrin	30	614.7 ± 12.3	57.0 ± 1.1	18.6 ± 0.4	39.6 ± 0.8	3 ± 0.1.1	2.3 ± 0.1
		40	489.9 ± 9.8	46.9 ± 0.9	26.5 ± 0.5	25.7 ± 0.5	1.7 ± 0.1	1.8 ± 0.1
		50	416.5 ± 8.3	41.0 ± 0.8	12.3 ± 0.2	21.6 ± 0.4	1.3 ± 0.1	1.0 ± 0.1
				POMACE				
FD	Inulin	30	2420.2 ± 48.4	787.4 ± 15.7	214.8 ± 4.3	282.9 ± 5.7	1.5 ± 0.1	4.9 ± 0.1
		40	2036.4 ± 40.7	742.5 ± 14.8	120.7 ± 2.4	233.1 ± 4.7	1.6 ± 0.1	5.0 ± 0.1
		50	1473.5 ± 29.5	570.8 ± 11.4	65.7 ± 1.3	172.3 ± 3.4	1.9 ± 0.1	2.9 ± 0.1
	Maltodextrin	30	2241.6 ± 44.8	701.7 ± 14.0	193.8 ± 3.9	256.2 ± 5.1	1.6 ± 0.1	4.9 ± 0.1
		40	1623.3 ± 32.5	517.2 ± 10.3	143.7 ± 2.9	185.3 ± 3.7	1.2 ± 0.1	4.6 ± 0.1
		50	1482.6 ± 29.7	496.5 ± 9.9	112.2 ± 2.2	171.6 ± 3.4	1.3 ± 0.1	3.6 ± 0.1
VD/50	Inulin	30	2037.8 ± 40.8	155.9 ± 3.1	71.8 ± 1.4	102.5 ± 2.0	1.6 ± 0.1	4.9 ± 0.1
		40	1475.8 ± 29.5	114.9 ± 2.3	53.2 ± 1.1	74.1 ± 1.5	1.2 ± 0.1	4.6 ± 0.1
		50	1347.8 ± 27.0	110.3 ± 2.2	41.6 ± 0.8	68.6 ± 1.4	1.3 ± 0.1	3.6 ± 0.1
	Maltodextrin	30	2200.2 ± 44.0	175.0 ± 3.5	79.6 ± 1.6	113.2 ± 2.3	1.5 ± 0.1	4.9 ± 0.1
		40	1851.3 ± 37.0	165.0 ± 3.3	44.7 ± 0.9	93.3 ± 1.9	1.6 ± 0.1	5.0 ± 0.1
		50	1339.6 ± 26.8	126.9 ± 2.5	24.4 ± 0.5	68.9 ± 1.4	1.9 ± 0.1	2.9 ± 0.1
VD/60	Inulin	30	1451.8 ± 29.0	132.9 ± 2.7	38.7 ± 0.8	75.8 ± 1.5	1.5 ± 0.1	4.6 ± 0.1
		40	1420.6 ± 28.4	126.4 ± 2.5	38.3 ± 0.8	72.9 ± 1.5	1.1 ± 0.1	4.2 ± 0.1
		50	720.9 ± 14.4	88.4 ± 1.8	12.4 ± 0.2	45.3 ± 0.9	1.3 ± 0.1	1.9 ± 0.1
	Maltodextrin	30	2336.4 ± 46.7	230.1 ± 4.6	50.9 ± 1.0	119.9 ± 2.4	1.1 ± 0.1	6.1 ± 0.1
		40	1718.5 ± 34.4	141.2 ± 2.8	55.5 ± 1.1	84.0 ± 1.7	1.0 ± 0.1	4.0 ± 0.1
		50	1140.8 ± 22.8	111.6 ± 2.2	22.6 ± 0.5	59.2 ± 1.2	1.5 ± 0.1	2.8 ± 0.1
				JUICE				
FD	Inulin	30	158.2 ± 3.2	38.6 ± 0.8	11.6 ± 0.2	24.1 ± 0.5	ND	ND

		40	106.9 ± 2.1	26.4 ± 0.5	9.8 ± 0.2	14.6 ± 0.3	ND	ND
		50	80.6 ± 1.6	29.2 ± 0.6	7.3 ± 0.1	16.1 ± 0.3	ND	ND
	Maltodextrin	30	131.1 ± 2.6	33.5 ± 0.7	11.2 ± 0.2	21.8 ± 0.4	ND	ND
		40	104.0 ± 2.1	29.3 ± 0.6	9.3 ± 0.2	15.9 ± 0.3	ND	ND
		50	89.2 ± 1.8	29.6 ± 0.6	6.4 ± 0.1	13.9 ± 0.3	ND	ND
VD/50	Inulin	30	116.8 ± 2.3	10.1 ± 0.2	4.2 ± 0.1	4.8 ± 0.1	ND	0.4 ± 0.01
		40	48.6 ± 1.1	3.0 ± 0.1	1.8 ± 0.1	1.1 ± 0.1	ND	0.4 ± 0.01
		50	44.3 ± 0.9	3.6 ± 0.1	1.1 ± 0.1	1.0 ± 0.1	ND	0.1 ± 0.01
	Maltodextrin	30	79.6 ± 1.6	7.2 ± 0.1	1.6 ± 0.1	3.4 ± 0.1	ND	0.4 ± 0.01
		40	75.1 ± 1.5	6.7 ± 0.1	1.9 ± 0.1	4.7 ± 0.1	ND	0.3 ± 0.01
		50	58.4 ± 1.2	5.3 ± 0.1	2.4 ± 0.1	3.0 ± 0.1	ND	0.2 ± 0.01
VD/60	Inulin	30	31.1 ± 0.6	4.5 ± 0.1	1.1 ± 0.1	2.3 ± 0.1	ND	0.5 ± 0.01
		40	19.6 ± 0.4	1.7 ± 0.1	0.7 ± 0.1	2.0 ± 0.1	ND	0.5 ± 0.01
		50	28.0 ± 0.6	3.2 ± 0.1	1.8 ± 0.1	1.2 ± 0.1	ND	0.2 ± 0.01
	Maltodextrin	30	78.9 ± 1.6	7.2 ± 0.1	4.0 ± 0.1	4.0 ± 0.1	ND	0.5 ± 0.01
		40	66.1 ± 1.3	6.5 ± 0.1	4.4 ± 0.1	3.0 ± 0.1	ND	0.3 ± 0.01
		50	47.4 ± 0.9	5.6 ± 0.1	1.3 ± 0.1	2.7 ± 0.1	ND	0.4 ± 0.01

[1] Values are expressed as the mean (*n* = 3) ± standard deviation. ND, no detect; FD, freeze-drying; VD/50, vacuum-drying at 50 °C; VD/60, vacuum-drying at 60 °C; ANT, sum of anthocyanins; C-3-gal, cyanidin-3-O-galactoside; C-3-glu, cyanidin-3-*O*-glucoside; C-3-ara, cyanidin-3-*O*-arabinoside; C-3-xyl, cyanidin-3-*O*-xyloside; C, cyanidin.

Table 3. Identification and quantification of phenolic acids [mg/100 g d.m.] in the fruit, juice, and pomace powders made form Saskatoon berry.

Drying method	Type of carriers	Con. [%]	PrA	CH	ThA	3CQA	Cag	5CQA	4CQA	*3pCQA*	DCQA	DCQA
FRUIT												
FD	Inulin	30	7.2 ± 0.1	6.9 ± 0.1	23.2 ± 0.5	332.7 ± 6.7	74.3 ± 1.5	848.9 ± 17.0	208.3 ± 4.2	3.5 ± 0.1	9.4 ± 0.2	58 ± 1.2
		40	8.6 ± 0.2	6.3 ± 0.1	22.9 ± 0.5	293.3 ± 5.9	66.5 ± 1.3	611.7 ± 12.2	114.2 ± 2.3	1.8 ± 0.1	5.3 ± 0.1	44.9 ± 0.9
		50	9.4 ± 0.2	6.6 ± 0.1	14.3 ± 0.3	250.3 ± 5	57.7 ± 1.2	499.5 ± 10	76.9 ± 1.5	1.2 ± 0.1	4.6 ± 0.1	11.9 ± 0.2
	Maltodextrin	30	10.3 ± 0.2	5.9 ± 0.1	24.8 ± 0.5	340 ± 6.8	69.6 ± 1.4	817.8 ± 16.4	205.7 ± 4.1	2.9 ± 0.1	7.9 ± 0.2	20.5 ± 0.4
		40	9.1 ± 0.2	5.1 ± 0.1	18.7 ± 0.4	323.2 ± 6.5	71.3 ± 1.4	720.6 ± 14.4	166.4 ± 3.3	2.1 ± 0.1	5.5 ± 0.1	15.2 ± 0.3
		50	8.7 ± 0.2	5.4 ± 0.1	11.7 ± 0.2	205.5 ± 4.1	55.0 ± 1.1	460.7 ± 9.2	145 ± 2.9	1.4 ± 0.1	3.2 ± 0.1	10.5 ± 0.2
VD/50	Inulin	30	9.7 ± 0.2	7.8 ± 0.2	19.7 ± 0.4	339.0 ± 6.8	96.6 ± 1.9	971.5 ± 19.4	335.1 ± 6.7	4.4 ± 0.1	6.6 ± 0.1	40.9 ± 0.8
		40	10.1 ± 0.2	6.3 ± 0.1	10.9 ± 0.2	215.6 ± 4.3	65.2 ± 1.3	749.4 ± 15.0	206.4 ± 4.1	2.5 ± 0.1	4.0 ± 0.1	42.5 ± 0.8
		50	8.9 ± 0.2	5.2 ± 0.1	10.5 ± 0.2	170.1 ± 3.4	46.9 ± 0.9	565.7 ± 11.3	146.9 ± 2.9	1.8 ± 0.1	3.2 ± 0.1	21.2 ± 0.4
	Maltodextrin	30	10.3 ± 0.2	9.7 ± 0.2	17.6 ± 0.4	363.7 ± 7.3	92.3 ± 1.8	1050.0 ± 21.0	262.8 ± 5.3	3.4 ± 0.1	7.1 ± 0.1	88.6 ± 1.8
		40	10.5 ± 0.2	8.3 ± 0.2	12 ± 0.2	227.5 ± 4.6	81.5 ± 1.6	853.0 ± 17.1	233.0 ± 4.7	2.0 ± 0.1	4.1 ± 0.1	42.7 ± 0.9
		50	9.9 ± 0.2	6.2 ± 0.1	10.4 ± 0.2	158.6 ± 3.2	53.9 ± 1.1	534.1 ± 10.7	159.2 ± 3.2	1.5 ± 0.1	3.3 ± 0.1	26.4 ± 0.5
VD/60	Inulin	30	6.8 ± 0.1	8.9 ± 0.2	20.6 ± 0.4	361.1 ± 7.2	99.6 ± 2.0	911.2 ± 18.2	281 ± 5.6	5.3 ± 0.1	6.7 ± 0.1	42.1 ± 0.8
		40	6.1 ± 0.1	6.3 ± 0.1	14.4 ± 0.3	222.9 ± 4.5	72.6 ± 1.5	660 ± 13.2	252.5 ± 5.1	4.2 ± 0.1	3.6 ± 0.1	37.4 ± 0.7
		50	5.8 ± 0.1	5.0 ± 0.1	12.5 ± 0.3	164.1 ± 3.3	55.9 ± 1.1	460.4 ± 9.2	147.4 ± 2.9	1.4 ± 0.1	3.2 ± 0.1	12.9 ± 0.3
	Maltodextrin	30	9.8 ± 0.2	7.9 ± 0.2	15.8 ± 0.3	311.4 ± 6.2	103 ± 2.1	906.3 ± 18.1	268.5 ± 5.4	4.2 ± 0.1	6.8 ± 0.1	75.4 ± 1.5
		40	7.8 ± 0.2	8.6 ± 0.2	12.3 ± 0.2	200.6 ± 4.0	84.7 ± 1.7	608.9 ± 12.2	226.4 ± 4.5	3.7 ± 0.1	3.9 ± 0.1	43.8 ± 0.9
		50	6.2 ± 0.1	5.6 ± 0.1	10.2 ± 0.2	161.4 ± 3.2	58.4 ± 1.2	463.8 ± 9.3	178.4 ± 3.6	3 ± 0.1	3.6 ± 0.1	28.6 ± 0.6
POMACE												
FD	Inulin	30	10.0 ± 0.2	2.0 ± 0.1	38.4 ± 0.8	571.5 ± 11.4	36.7 ± 0.7	800.1 ± 16.0	290.1 ± 5.8	2.8 ± 0.1	4.3 ± 0.1	27.8 ± 0.6
		40	8.4 ± 0.2	1.7 ± 0.1	45.8 ± 0.9	474.5 ± 9.5	30.7 ± 0.6	684.2 ± 13.7	235.5 ± 4.7	2.5 ± 0.1	5.5 ± 0.1	24.2 ± 0.5
		50	8.0 ± 0.2	1.5 ± 0.1	47.8 ± 1.0	323.5 ± 6.5	27.6 ± 0.6	523.0 ± 10.5	201.1 ± 4.0	2.7 ± 0.1	6.7 ± 0.1	32.0 ± 0.6
	Maltodextrin	30	10.4 ± 0.2	1.2 ± 0.1	83.6 ± 1.7	369.9 ± 7.4	44.6 ± 0.9	896.7 ± 17.9	282.1 ± 5.6	3.0 ± 0.1	8.2 ± 0.2	31.2 ± 0.6
		40	9.9 ± 0.2	1.4 ± 0.1	61.0 ± 1.2	286.5 ± 5.7	31.4 ± 0.6	688.6 ± 13.8	249.6 ± 5.0	2.0 ± 0.1	5.5 ± 0.1	25.4 ± 0.5
		50	7.1 ± 0.1	1.4 ± 0.1	41.4 ± 0.8	259.0 ± 5.2	25.8 ± 0.5	455.8 ± 9.1	190.0 ± 3.8	1.3 ± 0.1	3.8 ± 0.1	15.5 ± 0.3
VD/50	Inulin	30	10.9 ± 0.2	2.2 ± 0.1	37.7 ± 0.8	358.2 ± 7.2	52.2 ± 1.0	717.4 ± 14.3	322.6 ± 6.5	3.1 ± 0.1	5.3 ± 0.1	51.4 ± 1.0
		40	10.1 ± 0.2	1.6 ± 0.1	27.3 ± 0.5	252.0 ± 5.0	36.3 ± 0.7	699.0 ± 14.0	236.3 ± 4.7	2.4 ± 0.1	3.5 ± 0.1	34.0 ± 0.7
		50	10.6 ± 0.2	1.7 ± 0.1	23.9 ± 0.5	237.2 ± 4.7	32.0 ± 0.6	684.4 ± 13.7	175.1 ± 3.5	1.6 ± 0.1	2.8 ± 0.1	31.7 ± 0.6
	Maltodextrin	30	10.6 ± 0.2	2.9 ± 0.1	39.1 ± 0.8	378.7 ± 7.6	54.2 ± 1.1	709.1 ± 14.2	302.2 ± 6.0	2.4 ± 0.1	5.1 ± 0.1	78.3 ± 1.6
		40	10.2 ± 0.2	2.9 ± 0.1	32.9 ± 0.7	303.7 ± 6.1	44.0 ± 0.9	663.2 ± 13.3	268.4 ± 5.4	1.7 ± 0.1	3.5 ± 0.1	58.2 ± 1.2
		50	10.0 ± 0.2	1.9 ± 0.1	23.5 ± 0.5	237.6 ± 4.8	32.2 ± 0.6	658.5 ± 13.2	225.2 ± 4.5	1.5 ± 0.1	3.1 ± 0.1	50.3 ± 1.0
VD/60	Inulin	30	10.9 ± 0.2	3.8 ± 0.1	19.2 ± 0.4	181.6 ± 3.6	59.8 ± 1.2	573.7 ± 11.5	383.2 ± 7.7	2.4 ± 0.1	2.5 ± 0.1	56.1 ± 1.1
		40	10.8 ± 0.2	2.4 ± 0.1	18.5 ± 0.4	171.8 ± 3.4	51.5 ± 1.0	554.8 ± 11.1	283.4 ± 5.7	2.4 ± 0.1	2.4 ± 0.1	40.9 ± 0.8
		50	10.5 ± 0.2	2.1 ± 0.1	17.4 ± 0.3	161.4 ± 3.2	58.4 ± 1.2	530.3 ± 10.6	260.6 ± 5.2	2.1 ± 0.1	2.3 ± 0.1	51.5 ± 1.0
	Maltodextrin	30	11.3 ± 0.2	3.1 ± 0.1	29.6 ± 0.6	282.2 ± 5.6	70.3 ± 1.4	569.5 ± 11.4	303.1 ± 6.1	3.1 ± 0.1	3.9 ± 0.1	69.0 ± 1.4
		40	10.9 ± 0.2	4.3 ± 0.1	23.6 ± 0.5	206.0 ± 4.1	65.7 ± 1.3	547.2 ± 10.9	261.5 ± 5.2	2.2 ± 0.1	2.7 ± 0.1	42.3 ± 0.8
		50	10.8 ± 0.2	2.3 ± 0.1	18.3 ± 0.4	146.2 ± 2.9	40.8 ± 0.8	525.0 ± 10.5	252.0 ± 5.0	1.6 ± 0.1	1.7 ± 0.1	49.6 ± 1.0
JUICE												
FD	Inulin	30	9.1 ± 0.2	5.5 ± 0.1	46.7 ± 0.9	360.5 ± 7.2	86.8 ± 1.7	654.1 ± 13.1	211.2 ± 4.2	2.1 ± 0.1	9.0 ± 0.2	15.5 ± 0.3

		40	9.7 ± 0.2	4.3 ± 0.1	29.3 ± 0.6	230.9 ± 4.6	57.1 ± 1.1	595.9 ± 11.9	132.7 ± 2.7	1.2 ± 0.1	6.5 ± 0.1	9.9 ± 0.2
		50	9.9 ± 0.2	6.6 ± 0.1	22.3 ± 0.4	164.5 ± 3.3	60.6 ± 1.2	488.1 ± 9.8	104.2 ± 2.1	1.9 ± 0.1	3.5 ± 0.1	6.2 ± 0.1
	Maltodextrin	30	9.3 ± 0.2	8.9 ± 0.2	43.3 ± 0.9	344.3 ± 6.9	96.3 ± 1.9	691.4 ± 13.8	202.5 ± 4.1	1.7 ± 0.1	8.5 ± 0.2	12.5 ± 0.3
		40	9.3 ± 0.2	7.4 ± 0.1	31.4 ± 0.6	244.7 ± 4.9	77.0 ± 1.5	609.5 ± 12.2	166.3 ± 3.3	1.4 ± 0.1	5.9 ± 0.1	9.6 ± 0.2
		50	9.3 ± 0.2	9.2 ± 0.2	20.3 ± 0.4	150.5 ± 3.0	56.2 ± 1.1	508.2 ± 10.2	111.6 ± 2.2	1.1 ± 0.1	3.2 ± 0.1	5.8 ± 0.1
VD/50	Inulin	30	8.7 ± 0.2	5.8 ± 0.1	32.5 ± 0.6	280.0 ± 5.6	72.1 ± 1.4	557.2 ± 11.1	272.7 ± 5.5	2.2 ± 0.1	7.3 ± 0.1	11.3 ± 0.2
		40	10 ± 0.2	6.2 ± 0.1	26.2 ± 0.5	203.3 ± 4.1	66.4 ± 1.3	529.7 ± 10.6	150.7 ± 3.0	2.2 ± 0.1	4.8 ± 0.1	8.6 ± 0.2
		50	9.7 ± 0.2	5.7 ± 0.1	19.2 ± 0.4	151.2 ± 3.0	57.1 ± 1.1	436.1 ± 8.7	135.4 ± 2.7	1.3 ± 0.1	3.8 ± 0.1	5.3 ± 0.1
	Maltodextrin	30	9.5 ± 0.2	7.0 ± 0.1	34.8 ± 0.7	295.7 ± 5.9	84.6 ± 1.7	594.4 ± 11.9	216.0 ± 4.3	1.8 ± 0.1	7.4 ± 0.1	11.9 ± 0.2
		40	9.9 ± 0.2	6.3 ± 0.1	27.2 ± 0.5	217.1 ± 4.3	75.1 ± 1.5	557.4 ± 11.1	186.4 ± 3.7	1.8 ± 0.1	5.1 ± 0.1	8.7 ± 0.2
		50	9.5 ± 0.2	4.9 ± 0.1	19.9 ± 0.4	158.1 ± 3.2	53.8 ± 1.1	446.5 ± 8.9	140.5 ± 2.8	1.1 ± 0.1	3.8 ± 0.1	6.4 ± 0.1
VD/60	Inulin	30	10.3 ± 0.2	11.3 ± 0.2	35.3 ± 0.7	259.9 ± 5.2	112.5 ± 2.3	472.5 ± 9.4	235.1 ± 4.7	2.3 ± 0.1	6.6 ± 0.1	9.8 ± 0.2
		40	10.2 ± 0.2	4.1 ± 0.1	21.6 ± 0.4	157.8 ± 3.2	50.4 ± 1.0	456.7 ± 9.1	200.4 ± 4.0	2.1 ± 0.1	4.7 ± 0.1	6.8 ± 0.1
		50	9.1 ± 0.2	5.4 ± 0.1	29.2 ± 0.6	217.7 ± 4.4	64.9 ± 1.3	377.1 ± 7.5	158.1 ± 3.2	1.9 ± 0.1	6.1 ± 0.1	9.2 ± 0.2
	Maltodextrin	30	10.4 ± 0.2	7.6 ± 0.2	42.1 ± 0.8	347.9 ± 7.0	104.1 ± 2.1	482.3 ± 9.6	251.4 ± 5.0	3.9 ± 0.1	7.4 ± 0.1	14.5 ± 0.3
		40	10.3 ± 0.2	6.4 ± 0.1	30.2 ± 0.6	244.6 ± 4.9	80.0 ± 1.6	486.3 ± 9.7	201.5 ± 4.0	2.6 ± 0.1	5.9 ± 0.1	10.2 ± 0.2
		50	10.1 ± 0.2	5.4 ± 0.1	19.5 ± 0.4	151.5 ± 3.0	74.1 ± 1.5	396.2 ± 7.9	157.2 ± 3.1	2.0 ± 0.0	3.9 ± 0.1	5.5 ± 0.1

[1] Values are expressed as the mean (n = 3) ± standard deviation. FD, freeze-drying; VD/50, vacuum-drying at 50 °C; VD/60, vacuum-drying at 60 °C; PA, sum of phenolic acids; PP, polymeric procyanidins; PrA, protocatechuic acid; Cag, caffeic acid glucoside; Chx, caffeoylhexose; ThA, trihydroxycinnamoylquinic acid isomers; 3CQA, 3-*O*-caffequinic acid); 5CQA, 5-*O*-caffequinic acid); 4CQA, 4-*O*-caffequinic acid); *3pCQA*, 3-*O*-*p*-coumaroylquinic acid; DCQA, di-caffeoylquinic acid; DCQA, di-caffeoylquinic acid.

Table 4. Identification and quantification of flavonols [mg/100 g d.m.] in the fruit, juice, and pomace powders made form Saskatoon berry.

Drying method	Type of carriers	Con. [%]	K-3-gal	Q-3-ara	K-3-glu	Q	Q-3-rut	Q-3-rob	Q-3-gal	Q-3-glu	Q-3-ara	Q-3-xyl	Q-3-6gal	Q-3-6glu	Qdhe
							FRUIT								
FD	Inulin	30	44.1 ± 0.9	3.4 ± 0.1	2.3 ± 0.1	0.2 ± 0.01	44.9 ± 0.9	0.2 ± 0.01	210.0 ± 4.2	10.3 ± 0.2	3.7 ± 0.1	1.9 ± 0.1	2.1 ± 0.1	6.5 ± 0.1	5.4 ± 0.1
		40	36.8 ± 0.7	2.7 ± 0.1	2.1 ± 0.1	0.2 ± 0.01	34.5 ± 0.7	0.2 ± 0.01	170.3 ± 3.4	8.2 ± 0.2	3.4 ± 0.1	1.4 ± 0.1	1.7 ± 0.1	5.2 ± 0.1	4.4 ± 0.1
		50	25.0 ± 0.5	1.3 ± 0.1	0.7 ± 0.1	0.1 ± 0.01	26.9 ± 0.5	0.1 ± 0.01	131.4 ± 2.6	6.5 ± 0.1	2.8 ± 0.1	1.1 ± 0.1	1.2 ± 0.1	3.5 ± 0.1	3.7 ± 0.1
	Maltodextrin	30	52.2 ± 1.0	3.7 ± 0.1	2.2 ± 0.1	0.1 ± 0.01	48.8 ± 1.0	0.3 ± 0.01	234.1 ± 4.7	11.4 ± 0.2	5.7 ± 0.1	2.0 ± 0.1	2.1 ± 0.1	8.0 ± 0.2	8.0 ± 0.2
		40	38.5 ± 0.8	2.7 ± 0.1	1.7 ± 0.1	0.1 ± 0.01	37.3 ± 0.7	0.2 ± 0.01	181.1 ± 3.6	8.6 ± 0.2	4.1 ± 0.1	1.5 ± 0.1	1.6 ± 0.1	6.5 ± 0.1	5.5 ± 0.1
		50	22.9 ± 0.5	1.6 ± 0.1	1.1 ± 0.0	0.1 ± 0.01	23.9 ± 0.5	0.2 ± 0.01	113.8 ± 2.3	5.7 ± 0.1	2.2 ± 0.1	1.1 ± 0.1	1.0 ± 0.1	4.0 ± 0.1	3.5 ± 0.1
VD/50	Inulin	30	26.3 ± 0.5	2.8 ± 0.1	2.8 ± 0.1	0.1 ± 0.01	45.4 ± 0.9	0.3 ± 0.01	200.0 ± 4.0	9.4 ± 0.2	3.2 ± 0.1	2.0 ± 0.1	1.6 ± 0.1	5.9 ± 0.1	12.7 ± 0.3
		40	23.2 ± 0.5	2.7 ± 0.1	1.9 ± 0.1	0.1 ± 0.01	29.3 ± 0.6	0.2 ± 0.01	147.3 ± 2.9	6.7 ± 0.1	1.3 ± 0.1	1.1 ± 0.1	1.1 ± 0.1	5.0 ± 0.1	11.1 ± 0.2
		50	17.6 ± 0.4	1.8 ± 0.1	1.2 ± 0.1	0.1 ± 0.01	23.1 ± 0.5	0.2 ± 0.01	115.4 ± 2.3	5.5 ± 0.1	2.0 ± 0.1	0.9 ± 0.1	1.0 ± 0.1	3.8 ± 0.1	7.1 ± 0.1
	Maltodextrin	30	30.3 ± 0.6	3.9 ± 0.1	3.9 ± 0.1	0.2 ± 0.01	46.2 ± 0.9	0.3 ± 0.01	207.8 ± 4.2	9.9 ± 0.2	2.7 ± 0.1	1.9 ± 0.1	1.7 ± 0.1	6.6 ± 0.1	14.2 ± 0.3
		40	19.5 ± 0.4	2.7 ± 0.1	2.2 ± 0.1	0.2 ± 0.01	31.0 ± 0.6	0.2 ± 0.01	148.1 ± 3.0	7.1 ± 0.1	2.5 ± 0.1	1.6 ± 0.1	1.2 ± 0.1	4.9 ± 0.1	9.6 ± 0.2
		50	13.9 ± 0.3	2.0 ± 0.1	1.3 ± 0.1	0.2 ± 0.01	22.0 ± 0.4	0.1 ± 0.01	110.2 ± 2.2	5.3 ± 0.1	2.1 ± 0.1	1.0 ± 0.1	0.8 ± 0.1	3.3 ± 0.1	5.3 ± 0.1
VD/60	Inulin	30	29.8 ± 0.6	2.6 ± 0.1	3.0 ± 0.1	0.2 ± 0.01	44.0 ± 0.9	0.3 ± 0.01	208.1 ± 4.2	10.4 ± 0.2	4.3 ± 0.1	2.5 ± 0.1	1.8 ± 0.1	6.8 ± 0.1	8.3 ± 0.2
		40	19.6 ± 0.4	2.4 ± 0.1	2.4 ± 0.1	0.2 ± 0.01	29.6 ± 0.6	0.2 ± 0.01	145.0 ± 2.9	6.5 ± 0.1	3.2 ± 0.1	2.0 ± 0.1	1.2 ± 0.1	5.1 ± 0.1	8.7 ± 0.2
		50	15.2 ± 0.3	1.4 ± 0.1	1.4 ± 0.1	0.1 ± 0.01	23.2 ± 0.5	0.3 ± 0.01	112.1 ± 2.2	5.6 ± 0.1	2.1 ± 0.1	1.0 ± 0.1	0.9 ± 0.1	4.0 ± 0.1	4.6 ± 0.1
	Maltodextrin	30	24.6 ± 0.5	3.4 ± 0.1	4.1 ± 0.1	0.1 ± 0.01	41.5 ± 0.8	0.1 ± 0.01	208.7 ± 4.2	10.1 ± 0.2	3.1 ± 0.1	1.8 ± 0.1	1.7 ± 0.1	7.0 ± 0.1	16.3 ± 0.3
		40	17.3 ± 0.3	2.0 ± 0.1	1.9 ± 0.1	0.1 ± 0.01	29.1 ± 0.6	0.1 ± 0.01	145.5 ± 2.9	7.0 ± 0.1	3.2 ± 0.1	1.3 ± 0.1	1.0 ± 0.1	4.5 ± 0.1	9.6 ± 0.2
		50	13.7 ± 0.3	1.9 ± 0.1	1.5 ± 0.1	0.1 ± 0.01	23.0 ± 0.5	0.2 ± 0.01	115.6 ± 2.3	5.7 ± 0.1	2.9 ± 0.1	1.2 ± 0.1	0.8 ± 0.1	3.6 ± 0.1	6.4 ± 0.1
							POMACE								
FD	Inulin	30	106.4 ± 2.1	5.2 ± 0.1	6.5 ± 0.1	ND	35.4 ± 0.7	0.4 ± 0.01	489.1 ± 9.8	23.1 ± 0.5	9.9 ± 0.2	3.5 ± 0.1	3.3 ± 0.1	16.4 ± 0.3	11.1 ± 0.2
		40	90.0 ± 1.8	4.9 ± 0.1	4.5 ± 0.1	ND	31.1 ± 0.6	0.3 ± 0.01	409.3 ± 8.2	19.7 ± 0.4	8.9 ± 0.2	2.9 ± 0.1	2.4 ± 0.1	14.2 ± 0.3	8.3 ± 0.2
		50	69.1 ± 1.4	3.2 ± 0.1	2.5 ± 0.1	ND	22.5 ± 0.4	0.2 ± 0.01	463.1 ± 9.3	14.2 ± 0.3	5.7 ± 0.1	2.4 ± 0.1	2.0 ± 0.1	10.1 ± 0.2	7.1 ± 0.1
	Maltodextrin	30	132.8 ± 2.7	8.8 ± 0.2	8.1 ± 0.2	0.3 ± 0.01	42.6 ± 0.9	0.4 ± 0.01	515.7 ± 10.3	25.3 ± 0.5	10.3 ± 0.2	4.2 ± 0.1	2.6 ± 0.1	20.4 ± 0.4	18.6 ± 0.4
		40	11.00 ± 2.2	5.9 ± 0.1	5.9 ± 0.1	0.2 ± 0.01	32.7 ± 0.7	0.3 ± 0.01	397.8 ± 8.0	19.8 ± 0.4	8.2 ± 0.2	3.5 ± 0.1	2.3 ± 0.1	14.5 ± 0.3	13.5 ± 0.3
		50	81.1 ± 1.6	3.5 ± 0.1	4.2 ± 0.1	0.1 ± 0.01	25.2 ± 0.5	0.2 ± 0.01	278.9 ± 5.6	14.1 ± 0.3	5.6 ± 0.1	2.3 ± 0.1	1.2 ± 0.1	9.9 ± 0.2	5.3 ± 0.1
VD/50	Inulin	30	69.5 ± 1.4	5.3 ± 0.1	2.6 ± 0.1	0.2 ± 0.01	50.6 ± 1	0.5 ± 0.01	348.3 ± 7.0	17.2 ± 0.3	8.1 ± 0.2	2.7 ± 0.1	3.2 ± 0.1	11.5 ± 0.2	15.5 ± 0.3
		40	49.0 ± 1.0	4.2 ± 0.1	2.9 ± 0.1	0.2 ± 0.01	36.2 ± 0.7	0.4 ± 0.01	257.0 ± 5.1	12.6 ± 0.3	5.3 ± 0.1	2.2 ± 0.1	2.3 ± 0.1	8.3 ± 0.2	10.5 ± 0.2
		50	40.6 ± 0.8	4.3 ± 0.1	3.6 ± 0.1	0.1 ± 0.01	30.0 ± 0.6	0.2 ± 0.01	217.2 ± 4.3	10.5 ± 0.2	4.6 ± 0.1	1.8 ± 0.1	2.1 ± 0.1	7.4 ± 0.1	7.9 ± 0.2
	Maltodextrin	30	76.4 ± 1.5	7.0 ± 0.1	4.5 ± 0.1	0.1 ± 0.01	48.5 ± 1.0	0.4 ± 0.01	333.5 ± 6.7	16.2 ± 0.3	6.4 ± 0.1	3.4 ± 0.1	3.2 ± 0.1	11.4 ± 0.2	14.1 ± 0.3
		40	62.6 ± 1.3	5.8 ± 0.1	2.4 ± 0.1	0.1 ± 0.01	38.7 ± 0.8	0.3 ± 0.01	270.3 ± 5.4	13.2 ± 0.3	8.2 ± 0.2	2.4 ± 0.1	2.5 ± 0.1	8.8 ± 0.2	16.0 ± 0.3
		50	42.2 ± 0.8	3.6 ± 0.1	1.9 ± 0.1	0.1 ± 0.01	30.7 ± 0.6	0.2 ± 0.01	206.0 ± 4.1	10.4 ± 0.2	4.2 ± 0.1	2.0 ± 0.1	1.8 ± 0.1	7.0 ± 0.1	9.5 ± 0.2
VD/60	Inulin	30	48.6 ± 1.0	8.4 ± 0.2	2.3 ± 0.1	0.1 ± 0.01	68.0 ± 1.4	0.7 ± 0.01	287.4 ± 5.7	13.1 ± 0.3	4.4 ± 0.1	2.4 ± 0.1	5.4 ± 0.1	9.3 ± 0.2	15.0 ± 0.3
		40	44.5 ± 0.9	7.3 ± 0.1	1.6 ± 0.1	0.1 ± 0.01	59.3 ± 1.2	0.4 ± 0.01	251.1 ± 5.0	11.9 ± 0.2	6.1 ± 0.1	2.1 ± 0.1	4.0 ± 0.1	7.6 ± 0.2	14.3 ± 0.3
		50	25.0 ± 0.5	5.1 ± 0.1	2.2 ± 0.1	0.1 ± 0.01	42.7 ± 0.9	0.3 ± 0.01	162.7 ± 3.3	6.9 ± 0.1	2.6 ± 0.1	1.5 ± 0.1	2.5 ± 0.1	4.3 ± 0.1	15.7 ± 0.3
	Maltodextrin	30	78.2 ± 1.6	11.2 ± 0.2	4.7 ± 0.1	0.1 ± 0.01	75.8 ± 1.5	0.3 ± 0.01	340.6 ± 6.8	16.0 ± 0.3	6.0 ± 0.1	2.8 ± 0.1	4.1 ± 0.1	10.9 ± 0.2	16.2 ± 0.3
		40	56.2 ± 1.1	8.5 ± 0.2	3.5 ± 0.1	0.1 ± 0.01	58.6 ± 1.2	0.3 ± 0.01	258.5 ± 5.2	12.0 ± 0.2	7.3 ± 0.1	2.1 ± 0.1	3.4 ± 0.1	8.3 ± 0.2	16.2 ± 0.3
		50	35.6 ± 0.7	6.2 ± 0.1	1.4 ± 0.1	0.1 ± 0.01	40.3 ± 0.8	0.2 ± 0.01	197.8 ± 4.0	9.7 ± 0.2	5.4 ± 0.1	2.0 ± 0.1	2.5 ± 0.1	6.9 ± 0.1	10.2 ± 0.2
							JUICE								
FD	Inulin	30	10.2 ± 0.2	1.9 ± 0.1	0.5 ± 0.01	0.1 ± 0.01	33.5 ± 0.7	0.1 ± 0.01	95.1 ± 1.9	4.8 ± 0.1	3.0 ± 0.1	1.4 ± 0.1	0.6 ± 0.01	3.6 ± 0.1	2.5 ± 0.1

		40	6.1 ± 0.1	2.0 ± 0.1	0.1 ± 0.01	0.1 ± 0.01	19.7 ± 0.4	0.2 ± 0.01	62.2 ± 1.2	5.4 ± 0.1	1.9 ± 0.1	1.1 ± 0.1	0.5 ± 0.01	2.6 ± 0.1	1.9 ± 0.1
		50	4.7 ± 0.1	0.3 ± 0.1	0.0 ± 0.00	0.1 ± 0.01	26.7 ± 0.5	0.1 ± 0.01	48.1 ± 1.0	2.6 ± 0.1	1.6 ± 0.1	0.9 ± 0.1	0.2 ± 0.01	0.4 ± 0.01	1.5 ± 0.1
	Maltodextrin	30	9.0 ± 0.2	1.6 ± 0.1	1.7 ± 0.1	0.1 ± 0.01	39.1 ± 0.8	0.1 ± 0.01	91.9 ± 1.8	4.7 ± 0.1	2.3 ± 0.1	1.2 ± 0.1	0.7 ± 0.01	3.8 ± 0.1	2.7 ± 0.1
		40	6.6 ± 0.1	1.8 ± 0.1	0.3 ± 0.01	0.1 ± 0.01	28.0 ± 0.6	0.2 ± 0.01	66.8 ± 1.3	3.5 ± 0.1	2.2 ± 0.1	1.0 ± 0.1	0.4 ± 0.01	2.8 ± 0.1	1.4 ± 0.1
		50	4.4 ± 0.1	0.9 ± 0.1	0.3 ± 0.01	0.1 ± 0.01	18.0 ± 0.4	0.2 ± 0.01	47.3 ± 0.9	2.6 ± 0.1	1.8 ± 0.1	0.9 ± 0.1	0.2 ± 0.01	1.9 ± 0.1	0.8 ± 0.1
VD/50	Inulin	30	8.7 ± 0.2	1.9 ± 0.1	0.5 ± 0.01	0.6 ± 0.01	32.4 ± 0.6	0.2 ± 0.01	98.3 ± 2.0	5.0 ± 0.1	2.8 ± 0.1	1.3 ± 0.1	0.7 ± 0.01	3.2 ± 0.1	3.8 ± 0.1
		40	5.1 ± 0.1	2.1 ± 0.1	1.3 ± 0.1	0.4 ± 0.01	20.3 ± 0.4	0.6 ± 0.01	72.9 ± 1.5	5.9 ± 0.1	2.2 ± 0.1	0.9 ± 0.1	0.4 ± 0.01	2.5 ± 0.1	2.5 ± 0.1
		50	3.8 ± 0.1	1.0 ± 0.1	0.0 ± 0.00	0.4 ± 0.01	19.0 ± 0.4	0.1 ± 0.01	54.3 ± 1.1	2.7 ± 0.1	1.9 ± 0.1	0.8 ± 0.1	0.8 ± 0.01	2.0 ± 0.1	1.8 ± 0.1
	Maltodextrin	30	8.1 ± 0.2	1.3 ± 0.1	0.2 ± 0.01	0.4 ± 0.01	33.9 ± 0.7	0.1 ± 0.01	96.8 ± 1.9	4.9 ± 0.1	2.3 ± 0.1	1.3 ± 0.1	0.8 ± 0.01	3.3 ± 0.1	4.5 ± 0.1
		40	5.2 ± 0.1	2.4 ± 0.1	0.4 ± 0.01	0.3 ± 0.01	25.9 ± 0.5	0.2 ± 0.01	76.1 ± 1.5	5.7 ± 0.1	1.5 ± 0.1	1.0 ± 0.1	0.5 ± 0.01	2.5 ± 0.1	2.5 ± 0.1
		50	4.2 ± 0.1	0.9 ± 0.1	0.3 ± 0.01	0.2 ± 0.01	18.8 ± 0.4	0.1 ± 0.01	54.8 ± 1.1	2.6 ± 0.1	1.2 ± 0.1	0.8 ± 0.1	0.3 ± 0.01	2.1 ± 0.1	1.4 ± 0.1
VD/60	Inulin	30	5.9 ± 0.1	1.1 ± 0.1	0.4 ± 0.01	0.4 ± 0.01	41.0 ± 0.8	0.3 ± 0.01	100.6 ± 2	4.5 ± 0.1	2.5 ± 0.1	1.5 ± 0.1	0.7 ± 0.01	2.9 ± 0.1	3.8 ± 0.1
		40	3.6 ± 0.1	2.6 ± 0.1	0.2 ± 0.01	0.3 ± 0.01	26.2 ± 0.5	0.4 ± 0.01	58.2 ± 1.2	5.4 ± 0.1	1.0 ± 0.1	0.9 ± 0.1	0.5 ± 0.01	1.9 ± 0.1	1.5 ± 0.1
		50	5.1 ± 0.1	1.5 ± 0.1	0.3 ± 0.01	0.3 ± 0.01	20.4 ± 0.4	0.3 ± 0.01	78.5 ± 1.6	3.9 ± 0.1	1.7 ± 0.1	1.5 ± 0.1	0.4 ± 0.01	3.2 ± 0.1	3.0 ± 0.1
	Maltodextrin	30	8.7 ± 0.2	1.6 ± 0.1	0.2 ± 0.01	0.3 ± 0.01	39.2 ± 0.8	0.2 ± 0.01	115.3 ± 2.3	5.4 ± 0.1	1.4 ± 0.1	1.5 ± 0.1	0.9 ± 0.01	3.2 ± 0.1	4.7 ± 0.1
		40	6.1 ± 0.1	2.9 ± 0.1	0.4 ± 0.01	0.2 ± 0.01	28.2 ± 0.6	0.3 ± 0.01	82.8 ± 1.7	5.9 ± 0.1	2.0 ± 0.1	1.2 ± 0.1	0.4 ± 0.01	2.5 ± 0.1	3.2 ± 0.1
		50	4.2 ± 0.1	1.1 ± 0.1	0.3 ± 0.01	0.2 ± 0.01	19.9 ± 0.4	0.1 ± 0.01	53.5 ± 1.1	2.5 ± 0.1	1.1 ± 0.1	0.7 ± 0.1	0.5 ± 0.01	1.7 ± 0.1	2.2 ± 0.1

[1] Values are expressed as the mean (n = 3) ± standard deviation. ND, no detect; FD, freeze-drying; VD/50, vacuum-drying at 50 °C; VD/60, vacuum-drying at 60 °C; FL, sum of flavonols; Q, quercetin; Q-3-rob, quercetin-3-*O*-robinobioside; Q-3-rut; quercetin-3-*O*-rutinoside; Q-3-ara; quercetin-3-*O*-arabinoside; Q-3-xyl, quercetin-3-*O*-xyloside; Q-3-glu, quercetin-3-*O*-glucoside; Q-3-gal, quercetin-3-*O*-galactoside; Q-3-arab; quercetin-3-*O*-arabinobioside; Q-3-6glu, quercetin-3-*O*-(6''-acetyl)glucoside; Q-3-6gal, quercetin-3-*O*-(6''-acetyl)galactoside; Qdhe, quercetin-deoxyhexo-heksoside; K-3-gal, kaempferol-3-*O*-galactoside; K-3-glu, kaempferol-3-*O*-glucoside.
